# A Population-Based Study: How to Identify High-Risk T1–2 Esophageal Cancer Patients?

**DOI:** 10.3389/fonc.2021.766181

**Published:** 2021-12-13

**Authors:** Yiming Qi, Shuangshuang Wu, Linghui Tao, Guoshu Xu, Jiabin Chen, Zhengquan Feng, Chao Lu, Yanli Wan, Jing Li

**Affiliations:** ^1^Department of Oncology, Tongde Hospital of Zhejiang Province, Hangzhou, China; ^2^Department of Geriatrics, Tongde Hospital of Zhejiang Province, Hangzhou, China; ^3^The Second Clinical Medical College of Zhejiang Chinese Medical University, Hangzhou, China; ^4^Department of Gastroenterology, The First Affiliated Hospital, Zhejiang University School of Medicine, Hangzhou, China; ^5^National Medicine Clinical Trial Organization Office, Tongde Hospital of Zhejiang Province, Hangzhou, China; ^6^Cancer Institute of Integrated Tradition Chinese and Western Medicine, Zhejiang Academy of Traditional Chinese Medicine, Tongde Hospital of Zhejiang Province, Hangzhou, China

**Keywords:** lymph node metastasis, distant metastasis, overall survival, nomogram, SEER database, T1–2 esophageal cancer

## Abstract

**Background:**

Due to individualized conditions of lymph node metastasis (LNM) and distant metastasis (DM), the following therapeutic strategy and diagnosis of T1–2 esophageal cancer (ESCA) patients are varied. A prediction model for identifying risk factors for LNM, DM, and overall survival (OS) of high-risk T1–2 ESCA patients is of great significance to clinical practice.

**Methods:**

A total of 1,747 T1–2 ESCA patients screened from the surveillance, epidemiology, and end results (SEER) database were retrospectively analyzed for their clinical data. Univariate and multivariate logistic regression models were established to screen out risk factors for LNM and DM of T1-2 ESCA patients, while those of OS were screened out using the Cox regression analysis. The identified risk factors for LNM, DM, and OS were then subjected to the establishment of three nomograms, respectively. The accuracy of the nomograms was evaluated by depicting the calibration curve, and the predictive value and clinical utility were evaluated by depicting the clinical impact curve (CIC) and decision curve analysis (DCA), respectively.

**Results:**

The age, race, tumor grade, tumor size, and T-stage were significant factors for predicting LNM of T1–2 ESCA patients (*p* < 0.05). The age, T-stage, tumor grade, and tumor size were significant factors for predicting DM of T1–2 ESCA patients (*p* < 0.05). The age, race, sex, histology, primary tumor site, tumor size, N-stage, M-stage, and surgery were significant factors for predicting OS of T1–2 ESCA patients (*p* < 0.05). The C-indexes of the three nomograms constructed by these factors were 0.737, 0.764, and 0.740, respectively, suggesting that they were clinically effective.

**Conclusions:**

The newly constructed nomograms can objectively and accurately predict the LNM, DM, and OS of T1–2 ESCA patients, which contribute to the individualized decision making before clinical management.

## Introduction

Esophageal cancer is a common malignant tumor of the digestive tract, with about 572,000 new cases and 508,000 deaths in 2018. Globally, ESCA ranks the 7th and 6th leading causes of cancer morbidity and mortality, respectively (1). According to the NCCN Guidelines for Esophageal and Esophagogastric Junction Cancers (Version 3. 2021), T1–2 ESCA has been defined to invade lamina propria, muscularis mucosae, submucosa, or muscularis propria, but not to invade fibrous membrane (2). For patients with newly diagnosed esophageal space-occupying lesions, their pathological diagnosis is often made by endoscopic biopsy (3). Most of T1–2 ESCA patients do not have LNM and DM at the initial diagnosis, but some of them suffer LNM and/or DM (4–6). Therapeutic strategies of ESCA are made according to individualized conditions of LNM and DM. For T1aN0M0 patients, only endoscopic mass resection is required, such as endoscopic submucosal dissection (ESD) (7), which is featured by a short length of stay, less complications, and high quality of life (8). However, early-stage ESCA is usually found during endoscopy, in which T-stage can be immediately judged, while LNM and DM cannot be clearly determined (9). LNM may occur after mass resection by endoscopy, and as a result, a following surgery is needed (10). Esophagectomy is recommended for cT1b-T2N0M0 ESCA patients, and neoadjuvant concurrent chemoradiotherapy plus esophagectomy is preferred to cT1b-T2N+M0 patients. The presence of DM significantly influences the clinical decision making, and therefore, LNM and DM should be monitored with the following examinations (2). Lymph node puncture can be performed when cervical LNM is suspected by clinical or ultrasound. Abdominal CT or MRI is performed for abdominal metastasis. Suspected metastases adjacent to the trachea and bronchus can be determined by ultrasonic bronchoscopy. PET-CT can be used to detect DM (2). In clinical practice, some gastroenterologists believed that T1–2 ESCA lesions do not break through the muscle layer, which are urgently resected under endoscopy. However, transferring to thoracic surgery once the endoscopic operation is unable to completely remove the tumor lesions can easily cause adverse consequences by empirical tumor resection and lymph node dissection under the circumstances where preoperative examinations are lacking. An early determination of LNM and DM of T1–2 ESCA patients based on their clinical data is beneficial to make individualized therapeutic strategies, reduce medical cost, and enhance the outcomes. In addition, the prognosis of T1–2 ESCA is largely influenced by LNM and DM. Therefore, predicting LNM and DM benefits the judgment of the prognosis of T1–2 ESCA earlier and more accurately.

The nomogram is an intuitive graphical prediction tool to calculate the risk of a clinical event in a patient (11). Compared with the widely used TNM staging system, the nomogram has better predictive ability for many malignant tumors (12). However, an accurate nomogram to predict LNM, DM, and OS in T1–2 ESCA patients is lacking. In this study, we intend to establish nomograms to predict LNM, DM, and OS of T1-2 ESCA patients by analyzing relevant clinical data in the SEER database.

## Methods

### Data Resources and Subjects

**Figure 1:**
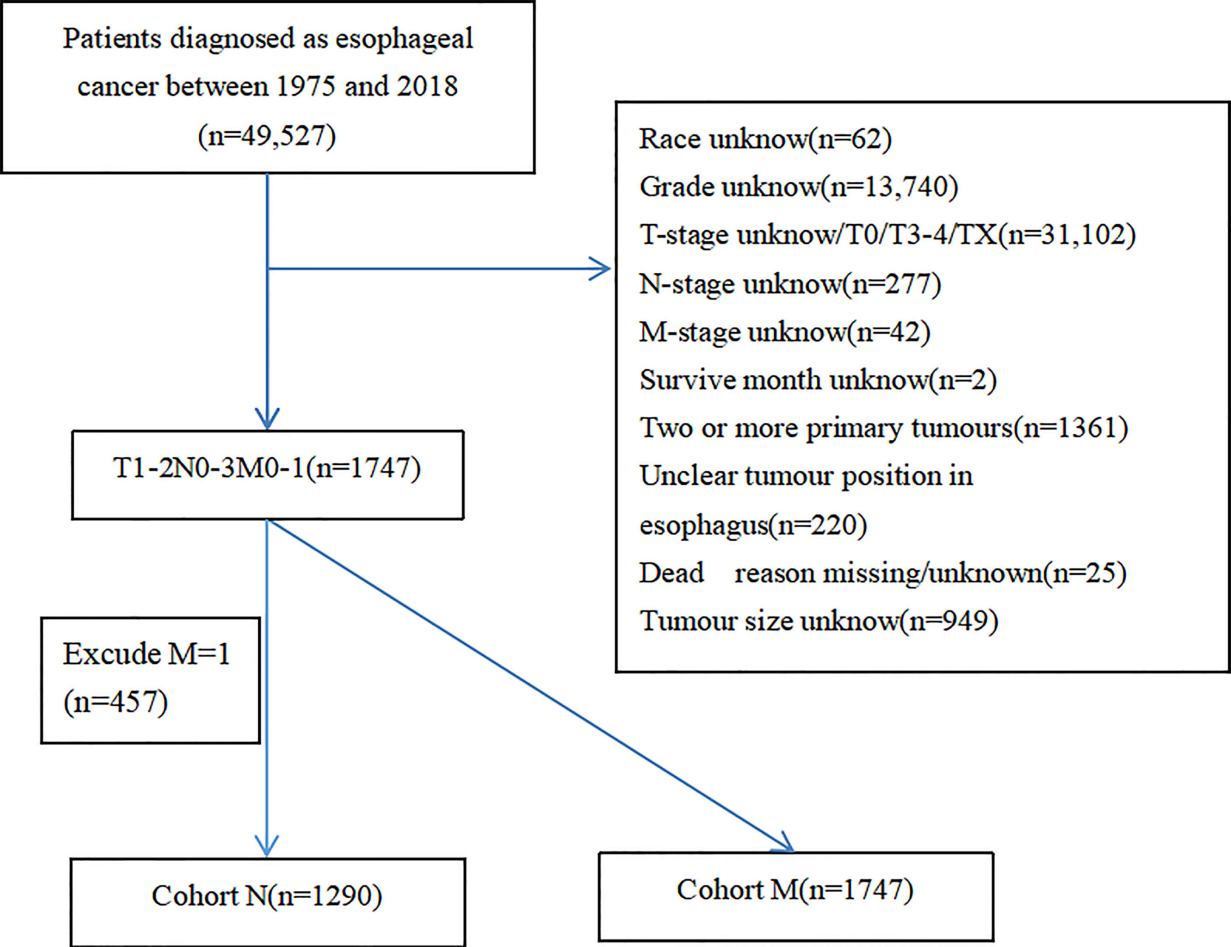
Case screening flow chart.

In this study, data of T1–2 ESCA patients were extracted from the SEER database, which is a publicly available database providing authorization information for cancer-related records of about 35% of the US population (13). Therefore, our research did not need ethical approval, with a large amount of data and guaranteed quality. A total of 49,527 T1–2 ESCA patients from 1975 to 2018 were obtained from the database. Exclusion criteria were as follows: (1) lack of clinical data the race, tumor grade, tumor position, and tumor size; (2) lack of survival data like vital status, survive time, and reason of death; (3) T0, T3–4, or unclear TNM staging (TX, NX, or MX); and (4) two or more primary tumors. Given the evidence that patients with DM are considered as advanced stage, lymph node status is not a decisive factor in the treatment (14). Recruited T1–2 ESCA patients were divided into group N (n = 1,290, T1–2M0 ESCA patients for predicting risk factors of LNM) and group M (n = 1,747, T1–2 ESCA patients for predicting risk factors of DM). Inclusion and exclusion criteria are shown in **Figure 1**.

### Variable Declaration

**Figure 2:**
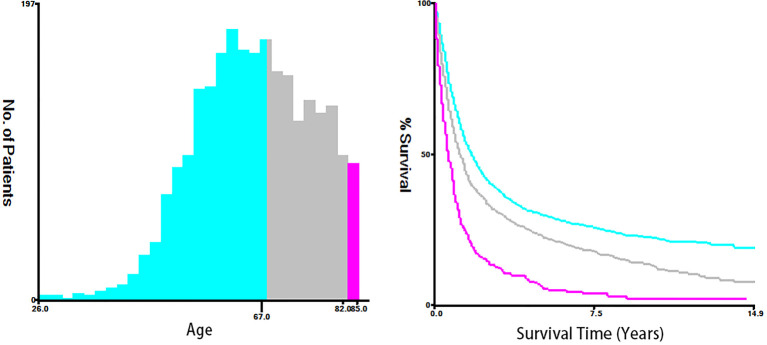
The optimal cutoff values were assessed by plotting Kaplan–Meier curves using the X-tile software. The age of T1–2 ESCA patients was categorized into ≤67, 68-81, and ≥82 years.

**Figure 3:**
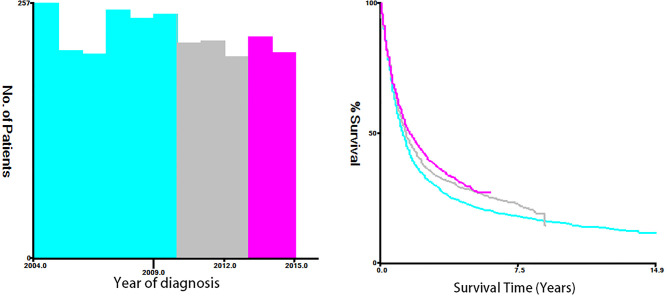
The optimal cutoff values were assessed by plotting Kaplan–Meier curves using the X-tile software. The year of diagnosis was categorized into 2004-2009, 2010-2012, and 2013-2015.

**Figure 4:**
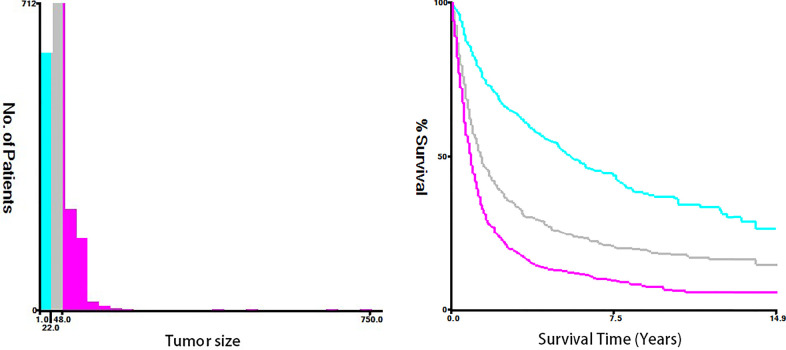
The optimal cutoff values were assessed by plotting Kaplan–Meier curves using the X-tile software. The tumor size of ESCA was categorized into 0-21, 22-47, and 48+ mm.

Fifteen clinicopathological variables were obtained from the SEER database, including the year of diagnosis, age, race, sex, tumor grade, histology, primary site, tumor size, T-stage, N-stage, M-stage, vital status, reason of death, surgery (primary site), and survival month. OS was defined as the span from the date of diagnosis to that of death from any cause. Cancer-specific survival (CSS) was defined as the time span from the date of diagnosis to that of death due to ESCA. For demographic variables, the optimal cutoff values for the year of diagnosis, age, and tumor size were assessed by plotting Kaplan–Meier curves using the X-tile software (Yale University, New Haven, Connecticut, USA) (15). Specifically, the year of diagnosis was categorized into 2004–2009, 2010–2012, and 2013–2015 (**Figure 2**). The age of T1–2 ESCA patients was categorized into ≤67, 68–81, and ≥82 years (**Figure 3**). The tumor size of ESCA was categorized into 0–21, 22–47, and 48+ mm (**Figure 4**). In addition, according to the arrangement of the SEER database and the needs of this study, other data were also classified. The pathological subtype of ESCA was categorized into adenocarcinoma, squamous cell carcinoma, and others according to the International Classification of Disease for Oncology 3^rd^ Edition, (ICD-O-3) hist/behav, malignant. According to the primary site labeled in SEER, the tumor site of ESCA was categorized into cervical esophagus, thoracic esophagus, abdominal esophagus, and overlapping lesion of esophagus. Since different AJCC versions were used for diagnosis, we carefully compared the 6th, 7th, and 8th, edition AJCC staging, and finally the 8th edition was adopted as follows: T1a/T1b was merged into T1, T4A/T4b (7th and 8th edition AJCC) was merged into T4, and N1–3 were merged into N+. The above modifications would not affect the accuracy of the research results.

### Nomogram Construction

We established univariate and multivariate logistic regression models (16) to screen out risk factors for LNM in group N and DM in group M, respectively. The Cox regression model was introduced to screen out prognostic factors of T1–2 ESCA. The effects of various factors on LNM, DM, and OS of T1–2 ESCA were measured by calculating the odds ratio (OR) and hazard ratio (HR). The subdistribution hazard region (SHR) was used to measure the impact of prognostic variables on CSS. The OS curve was drawn by the Kaplan–Meier method, and the cumulative incidence rate of tumor was plotted by cumulative incidence rate function. Then, two nomograms were created to predict the risk factors of LNM and DM in T1–2 ESCA patients according to the results of logistic regression models. According to the Cox proportional hazard model, a predictive nomogram was established to calculate the OS of T1–2 ESCA patients. These nomograms were validated by ROC and calibration curves for their accuracy. The C-index was used to reflect the accuracy of the model, in which a maximum of 1.0 indicated the perfect differentiation ability, and greater than 0.7 indicated a high accuracy of the prediction model. DCA, as a tool to evaluate the clinical application value of the nomogram (17), was used to evaluate the net benefit in this study. In addition, we plotted the CIC to reveal the value of the nomogram model more intuitively.

### Statistical Analysis

The optimal cutoff values for the age and tumor size in Kaplan–Meier survival curves were assessed by the X-tile software. The baseline of patients between the training group and the test group was tested through the chi-square test. The baseline characteristics of T1–2 ESCA patients were analyzed using SPSS 26.0 and *p* < 0.05 considered as statistically significant. Other data analyses were carried out through the corresponding functions of R software (version 4.0.3).

## Results

### Clinical Features of T1–2 ESCA

After a strict screening, 1,747 patients diagnosed with T1–2 ESCA between 2004 and 2015 were finally included in this study. They were divided into group N (T1-2N0-1M0, n = 1,290) and group M (T1N0-3M0-1, n = 1,747). The ratio of T1-2 ESCA patients with LNM in group N was 33.41% and that of DM in group M was 26.16%. Clinical data of recruited T1-2 ESCA patients are listed in **Tables 1**, **2**.

**Table 1:** Characteristics of patients with T1-2 ESCA (T1-2N0-3M0).

Characteristic	Nt (%)	Ne (%)	Nne (%)	p
	1290	431(33.41)	859(66.59)	
**Age**				<0.001
≤67	664 (51.47)	253 (58.70)	411 (47.85)	
68-81	458 (35.50)	141 (32.71)	317 (36.90)	
≥82	168 (13.02)	37 (8.58)	131 (15.25)	
**Year of diagnosis**				0.6
2004–2009	651 (50.47)	212 (49.19)	439 (51.11)	
2010–2012	325 (25.19)	116 (26.91)	209 (24.33)	
2013–2015	314 (24.34)	103 (23.90)	211 (24.56)	
**T-stage**				<0.001
T1	834 (64.65)	204 (47.33)	630 (73.34)	
T2	456 (35.35)	227 (52.67)	229 (26.66)	
**Sex**				0.974
Male	993 (76.98)	332 (77.03)	661 (76.95)	
Female	297 (23.02)	99 (22.97)	198 (23.05)	
**Race**				0.051
White	1100 (85.27)	356 (82.60)	744 (86.61)	
Black	98 (7.60)	35 (8.12)	63 (7.33)	
Asian or Pacific Islander	85 (6.59)	39 (9.05)	46 (5.36)	
American Indian/Alaska Native	7 (0.54)	1 (0.23)	6 (0.70)	
**Grade**				<0.001
Grade I (well differentiated)	122 (9.46)	14 (3.25)	108 (12.57)	
Grade II (moderately differentiated)	627 (48.60)	198 (45.94)	429 (49.94)	
Grade III (poorly differentiated)	512 (39.69)	208 (48.26)	304 (35.39)	
Grade IV (undifferentiated)	29 (2.25)	11 (2.55)	18 (2.10)	
**Primary site**				0.399
Cervical esophagus	70 (5.43)	26 (6.03)	44 (5.12)	
Thoracic esophagus	288 (22.33)	106 (24.59)	182 (21.19)	
Abdominal esophagus	899 (69.69)	287 (66.59)	612 (71.25)	
Overlapping lesion of esophagus	33 (2.56)	12 (2.78)	21 (2.44)	
**Histology**				0.004
Adenocarcinoma	869 (67.36)	264 (61.25)	605 (70.43)	
Squamous cell carcinoma	384 (29.77)	153 (35.50)	231 (26.89)	
Others	37 (2.87)	14 (3.25)	23 (2.68)	
**Vital status**				<0.001
Dead of other reasons or alive	390 (30.23)	94 (21.81)	296 (34.46)	
Dead of cancer	900 (69.77)	337 (78.19)	563 (65.54)	
**Tumor size**				<0.001
0–21 mm	465 (36.05)	97 (22.51)	368 (42.84)	
22–47 mm	460 (35.66)	161 (37.35)	299 (34.81)	
>48 mm	365 (28.29)	173 (40.14)	192 (22.35)	
**Surgery (primary site)**				<0.001
None	555 (43.02)	218 (50.58)	337 (39.23)	
Yes	735 (56.98)	213 (49.42)	522 (60.77)	

**Table 2:** Characteristics of patients with T1-2 ESCA (T1-2N0-3M0-1).

Characteristic	Mt (%)	Me (%)	Mne (%)	p
	1747	457(26.16)	1290(73.84)	
**Age**				
≤67	940 (53.81)	276 (60.39)	664 (51.47)	0.001
68–81	604 (34.57)	146 (31.95)	458 (35.50)	
≥82	203 (11.62)	35 (7.66)	168 (13.02)	
**Year of diagnosis**				
2004–2009	873 (49.97)	222 (48.58)	651 (50.47)	0.139
2010–2012	428 (24.50)	103 (22.54)	325 (25.19)	
2013–2015	446 (25.53)	132 (28.88)	314 (24.34)	
**T-stage**				
T1	1187 (67.95)	353 (77.24)	834 (64.65)	<0.001
T2	560 (32.05)	104 (22.76)	456 (35.35)	
**Sex**				
Male	1372 (78.53)	379 (82.93)	993 (76.98)	0.008
Female	375 (21.47)	78 (17.07)	297 (23.02)	
**Race**				
White	1481 (84.77)	381 (83.37)	1100 (85.27)	0.678
Black	134 (7.67)	36 (7.88)	98 (7.60)	
Asian or Pacific Islander	121 (6.93)	36 (7.88)	85 (6.59)	
American Indian/Alaska Native	11 (0.63)	4 (0.88)	7 (0.54)	
**Grade**				
Grade I (well differentiated)	129 (7.38)	7 (1.53)	122 (9.46)	<0.001
Grade II (moderately differentiated)	790 (45.22)	163 (35.67)	627 (48.60)	
Grade III (poorly differentiated)	787 (45.05)	275 (60.18)	512 (39.69)	
Grade IV (undifferentiated)	41 (2.35)	12 (2.63)	29 (2.25)	
**Primary site**				
Cervical esophagus	90 (5.15)	20 (4.38)	70 (5.43)	0.003
Thoracic esophagus	358 (20.49)	70 (15.32)	288 (22.33)	
Abdominal esophagus	1247 (71.38)	348 (76.15)	899 (69.69)	
Overlapping lesion of esophagus	52 (2.98)	19 (4.16)	33 (2.56)	
**Histology**				
Adenocarcinoma	1183 (67.72)	314 (68.71)	869 (67.36)	0.266
Squamous cell carcinoma	508 (29.08)	124 (27.13)	384 (29.77)	
Others	56 (3.21)	19 (4.16)	37 (2.87)	
**Vital status**				
Dead of other reasons or alive	410 (23.47)	20 (4.38)	390 (30.23)	<0.001
Dead of cancer	1337 (76.53)	437 (95.62)	900 (69.77)	
**Tumor size**				
0–21 mm	508 (29.08)	43 (9.41)	465 (36.05)	<0.001
22–47 mm	595 (34.06)	135 (29.54)	460 (35.66)	
>48 mm	644 (36.86)	279 (61.05)	365 (28.29)	
**Surgery (primary site)**				
None	973 (55.70)	418 (91.47)	555 (43.02)	<0.001
Yes	774 (44.30)	39 (8.53)	735 (56.98)	

### Risk Factors and Nomogram of LNM

According to univariate and multivariate logistic regression models, LNM was found closely related to the age at diagnosis, race, tumor grade, tumor size, and T-stage, while it was not correlated with sex, primary site, and histology (**Table 3**). In particular, T1–2 ESCA patients with the oldest age (≥82 years) had a lowest risk of LNM (OR = 0.34, 95% CI = 0.22–0.52, *p* < 0.001), followed by those aged 68–81 years (OR = 0.65, 95% CI = 0.49–0.85, *p* = 0.002). T2 ESCA patients had a higher risk of LNM than those with T1 (OR = 2.83, 95% CI = 2.19–3.66, *p* < 0.001). In addition, Asian or Pacific Islanders had the highest risk of LNM compared with that of White (OR = 1.74, 95% CI = 1.04–2.89, *p* = 0.033). T1–2 ESCA patients with grade II (OR = 2.79, 95% CI = 1.55–5.37, *p* = 0.001), grade III (or = 4.06, 95% CI = 2.25–7.81, *p* < 0.001), and grade IV (OR = 3.25, 95% CI = 1.17–9.01, *p* = 0.023) had a higher risk of LNM than that of grade I. Compared with T1–2 ESCA patients with a tumor size of 0–21 mm, those with a tumor size of 22–47 mm (OR = 1.77, 95% CI = 1.29–2.44, *p* < 0.001), and > 48 mm (OR = 3.21, 95% CI = 2.31–4.49, *p* < 0.001) had a higher risk of LNM.

**Table 3:** Logistic regression analysis of the risk factors for LNM in cohort N.

Factors	Univariate analysis		Multivariate analysis	
Age	OR (95%)	p	OR (95%)	p
≤67	Reference		Reference	
68–81	0.72 (0.56–0.93)	0.012	0.65 (0.49–0.85)	0.002
≥82	0.46 (0.30–0.68)	<0.001	0.34 (0.22–0.52)	<0.001
**Year of diagnosis**				
2004–2009	Reference		Reference	
2010–2012	1.15 (0.87–1.52)	0.330	1.15 (0.84–1.56)	0.375
2013–2015	1.01 (0.76–1.34)	0.941	1.07 (0.78–1.47)	0.655
**T-stage**				
T1	Reference		Reference	
T2	3.06 (2.40–3.90)	<0.001	2.83 (2.19–3.66)	<0.001
**Sex**				
Male	Reference		Reference	
Female	1.00 (0.75–1.31)	0.974	0.93 (0.68–1.28)	0.667
**Race**				
White	Reference		Reference	
Black	1.16 (0.75–1.78)	0.498	0.85 (0.52–1.39)	0.527
Asian or Pacific Islander	1.77 (1.13–2.76)	0.012	1.74 (1.04–2.89)	0.033
American Indian/Alaska Native	0.35 (0.02–2.05)	0.330	0.31 (0.02–1.94)	0.290
**Grade**				
Grade I (well differentiated)	Reference		Reference	
Grade II (moderately differentiated)	3.56 (2.06–6.64)	<0.001	2.79 (1.55–5.37)	0.001
Grade III (poorly differentiated)	5.28 (3.04–9.86)	<0.001	4.06 (2.25–7.81)	<0.001
Grade IV (undifferentiated)	4.71 (1.84–12.08)	0.001	3.25 (1.17–9.01)	0.023
**Primary site**				
Cervical esophagus	Reference		Reference	
Thoracic esophagus	0.99 (0.58–1.71)	0.958	1.01 (0.57–1.82)	0.972
Abdominal esophagus	0.79 (0.48–1.33)	0.369	0.89 (0.50–1.61)	0.702
Overlapping lesion of esophagus	0.97 (0.40–2.27)	0.939	0.67 (0.26–1.69)	0.395
**Histology**				
Adenocarcinoma	Reference		Reference	
Squamous cell carcinoma	1.52 (1.18–1.95)	0.001	1.22 (0.84–1.78)	0.285
Others	1.39 (0.69–2.72)	0.337	1.15 (0.53–2.42)	0.723
**Tumor size**				
0–21 mm	Reference		Reference	
22–47 mm	2.04 (1.52–2.75)	<0.001	1.77 (1.29–2.44)	<0.001
>48 mm	3.42 (2.53–4.64)	<0.001	3.21 (2.31–4.49)	<0.001

**Figure 5:**
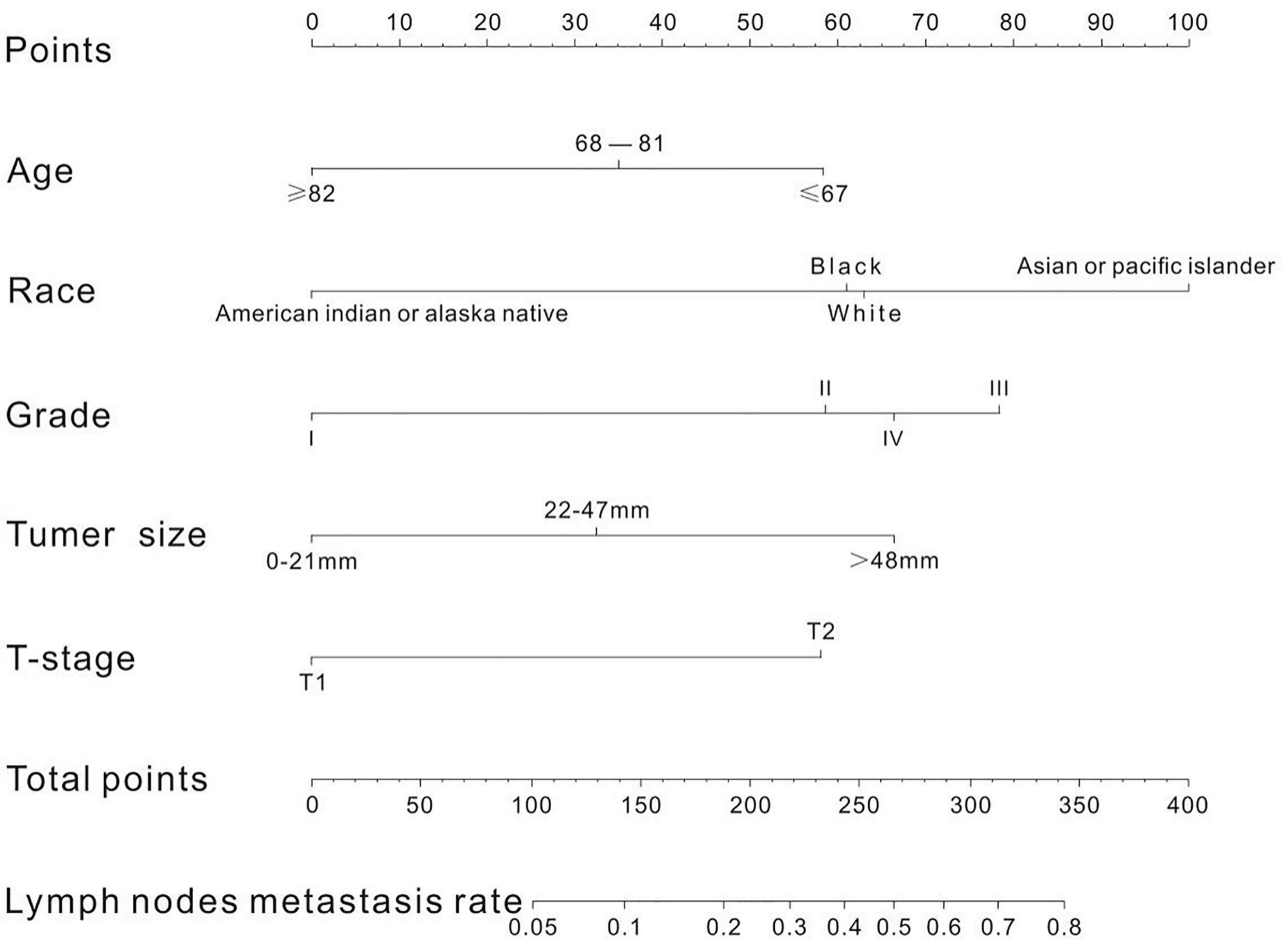
There are five factors in the nomogram. After taking values for these five factors (the upper scale), the total score is calculated, and the corresponding LNM rate is obtained according to the total score.

**Figure 6:**
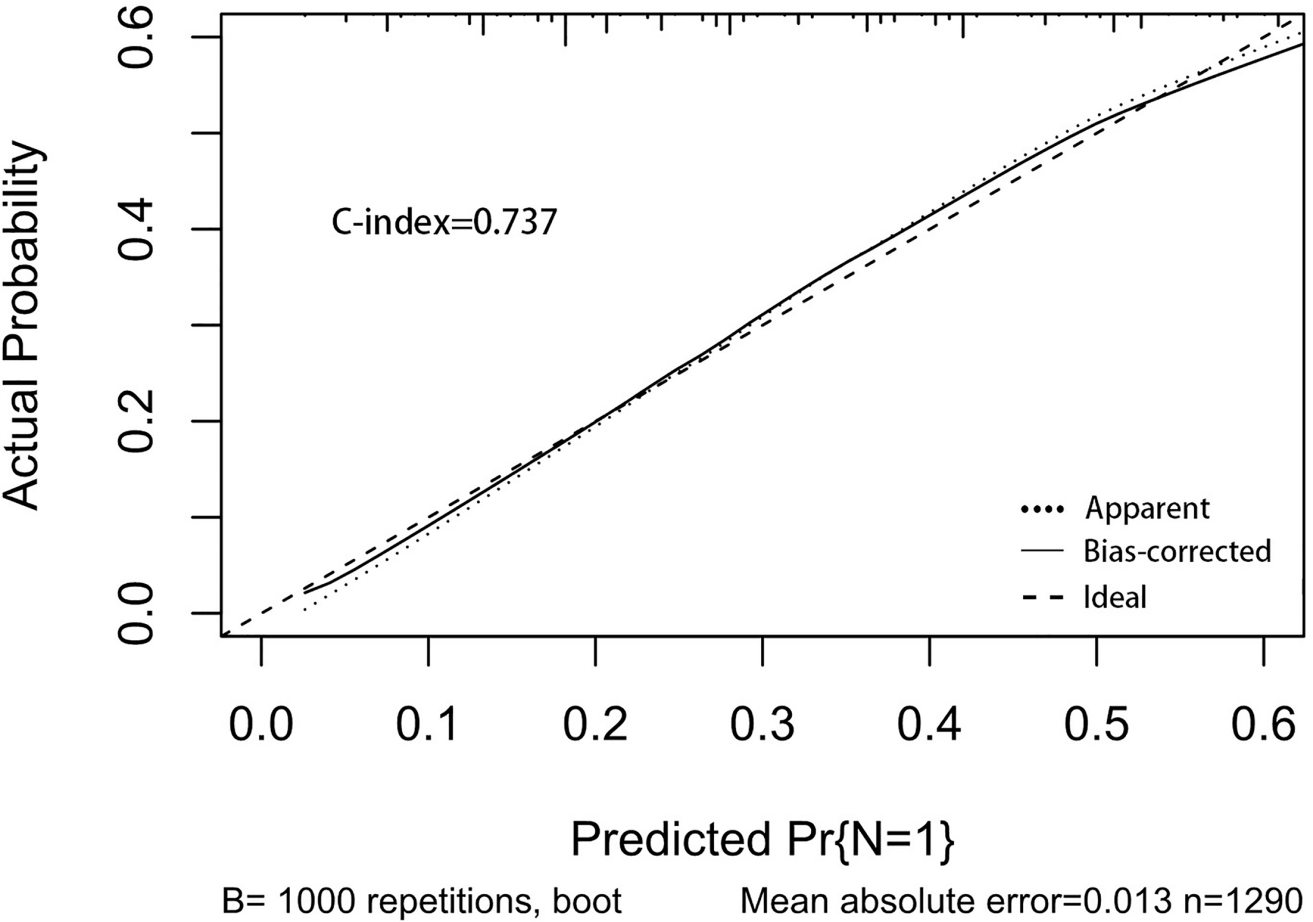
The calibration curve used to predict LNM, with C-index at 0.737. The diagonal line indicates a coincidence between the actual and predicted LNM probabilities, indicating that the probability predicted by the nomogram is very consistent with the actual observed values. The solid line is close to the diagonal line.

**Figure 7:**
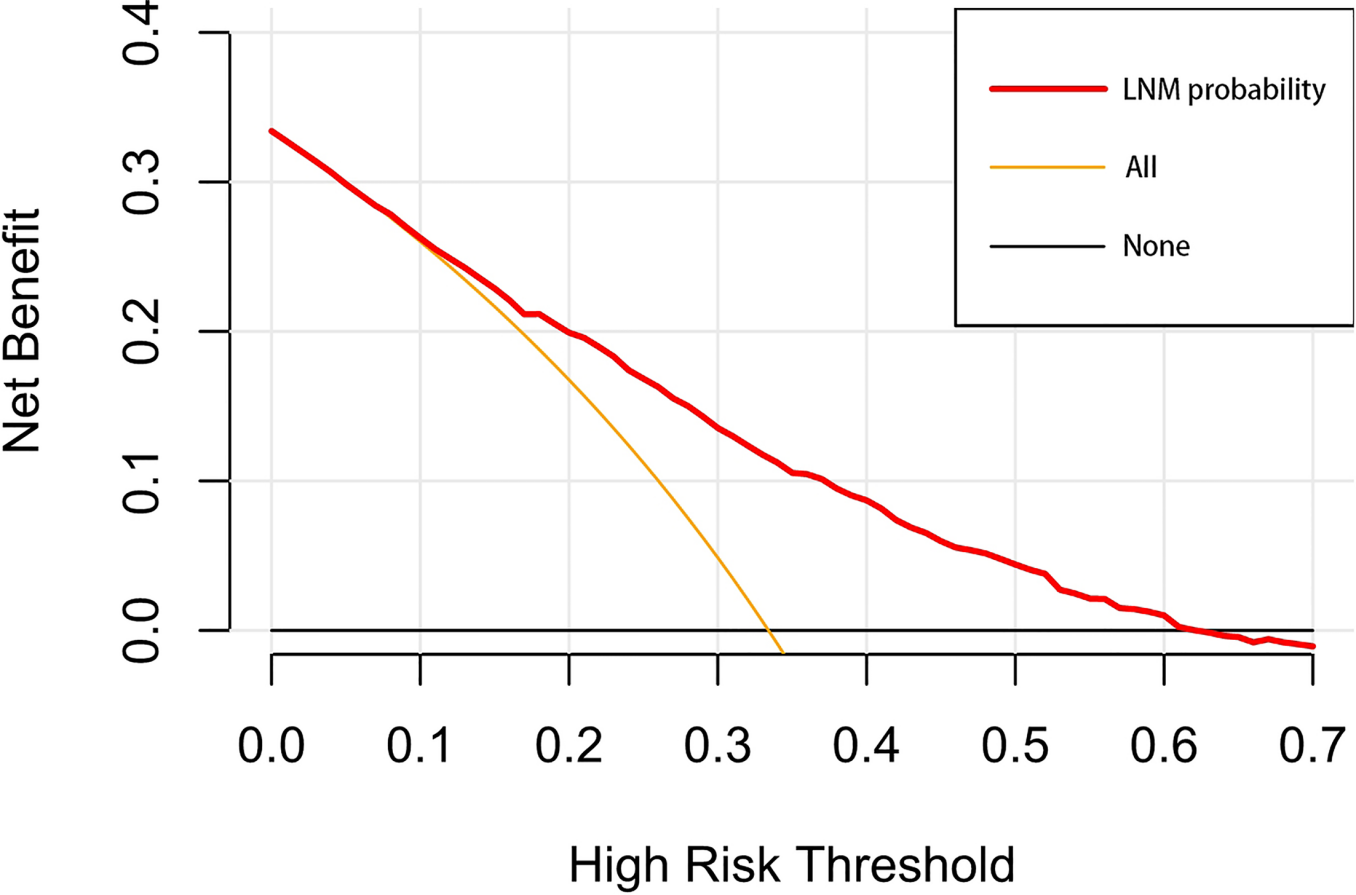
The decision curve draws a nomogram of the predicted LNM. The x-axis represents the threshold probability and the y-axis represents the net benefit. The horizontal black line indicates that no patient has an extreme condition of LNM, and the yellow line indicates that all patients have another extreme condition of LNM.

**Figure 8:**
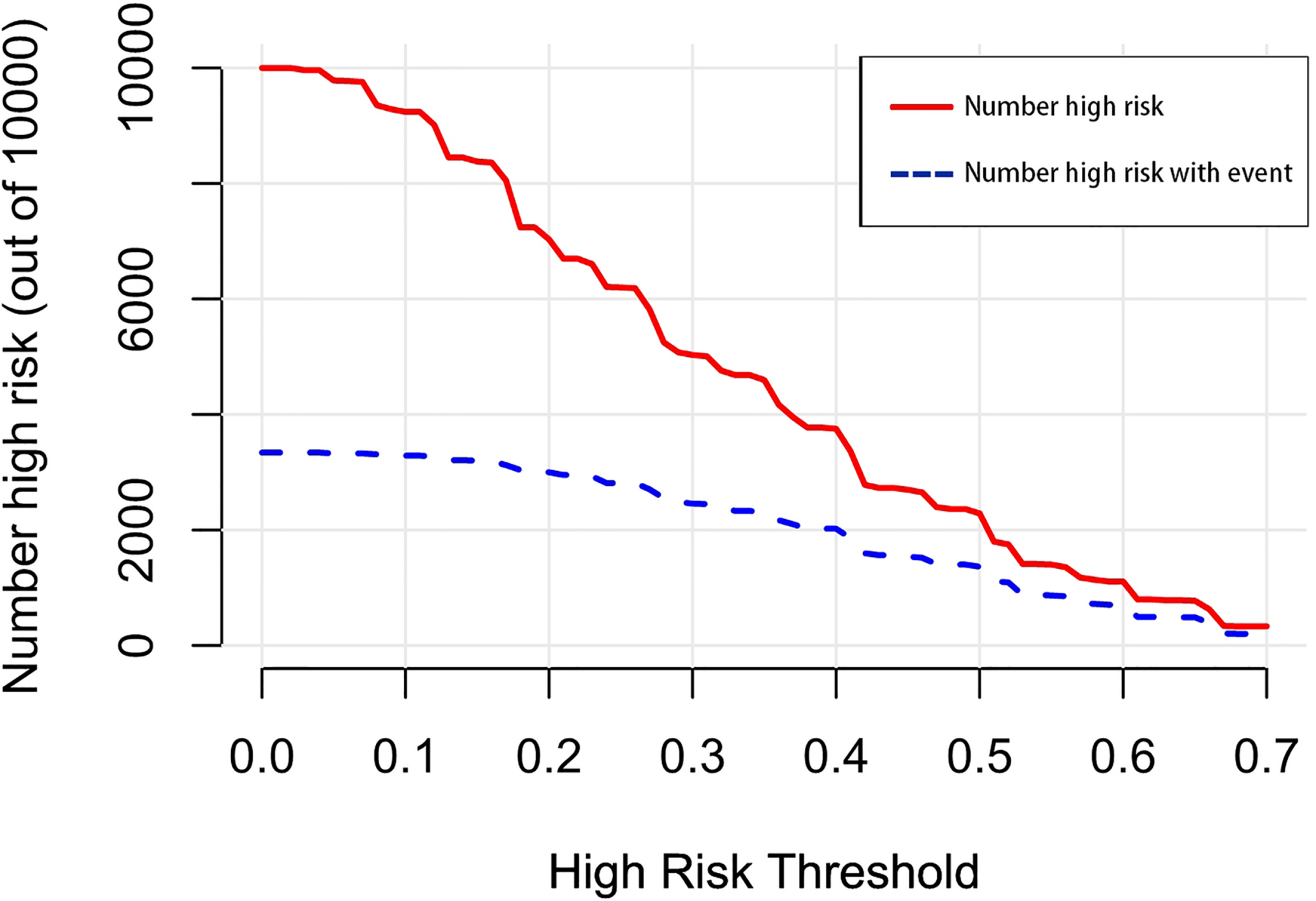
The number of high-risk patients and the number of high-risk patients with events are drawn with red solid lines and blue dotted lines to represent different threshold probabilities, respectively.

A nomogram was established to visually display the risk factors of LNM (**Figure 5**). In addition, the exact scores of each factor in the nomogram are as shown in **Table 5**. Ranked by the weight of each influencing factor, the race of T1–2 ESCA patients was on the top place, followed by tumor grade, tumor size, age, and T-stage. The calibration curve revealed that the nomogram had a strong resolution, and the C-index was 0.737 (**Figure 6**). In addition, a N-cohort study of DCA and CIC on the LNM nomogram was conducted, showing that our nomogram was favorable to predict LNM in T1–2 ESCA patients in the threshold range of 0–0.35 (**Figures 7**, **8**).

### Risk Factors and Nomogram of DM

According to univariate and multivariate logistic regression models, we found that DM was closely related to age, T-stage, tumor grade, and tumor size, while it was not correlated with sex, race, primary site, and histology (**Table 4**). The risk of DM in T1–2 ESCA patients with 68–81 years (OR = 0.72, 95% CI = 0.55–0.93, *p* = 0.013) and ≥82 years (OR = 0.41, 95% CI = 0.26–0.62, *p* < 0.001) was relatively low. Different from LNM, T2 ESCA patients were less prone to have DM than T1 patients (OR = 0.44, 95% CI = 0.33–0.57, *p* < 0.001). DM was more likely to affect grade IV (OR = 5.07, 95% CI = 1.75–15.63, *p* < 0.001), grade III (OR = 6.84, 95% CI = 3.27–16.75, *p* < 0.001), or grade II ESCA patients (OR = 3.77, 95% CI = 1.79–9.25, *p* = 0.001). In addition, T1–2 ESCA patients with a tumor size of 22–47 mm (OR = 3.34, 95% CI = 2.30–4.94, *p* < 0.001), and >48 mm (OR = 8.56, 95% CI = 5.98–12.51, *p* < 0.001) had a higher risk for DM than those with 0–21 mm.

**Table 4:** Logistic regression analysis of the risk factors for DM in cohort M.

Factors	Univariate analysis		Multivariate analysis	
Age	OR (95%)	p	OR(95%)	p
≤67	Reference		Reference	
68–81	0.77 (0.61–0.97)	0.026	0.72 (0.55–0.93)	0.013
≥82	0.5 (0.33–0.73)	<0.001	0.41 (0.26–0.62)	<0.001
**Year of diagnosis**				
2004–2009	Reference		Reference	
2010–2012	0.93 (0.71–1.21)	0.593	1.02 (0.76–1.37)	0.895
2013–2015	1.23 (0.95–1.59)	0.106	1.54 (1.15–2.05)	0.003
**T-stage**				
T1	Reference		Reference	
T2	0.54 (0.42–0.69)	<0.001	0.44 (0.33–0.57)	<0.001
**Sex**				
Male	Reference		Reference	
Female	0.69 (0.52–0.90)	0.008	0.90 (0.65–1.23)	0.514
**Race**				
White	Reference		Reference	
Black	1.06 (0.70–1.57)	0.773	1.00 (0.61–1.59)	0.985
Asian or Pacific Islander	1.22 (0.81–1.82)	0.332	1.23 (0.76–1.96)	0.398
American Indian/Alaska Native	1.65 (0.43–5.49)	0.427	0.81 (0.20–2.91)	0.753
**Grade**				
Grade I (well differentiated)	Reference		Reference	
Grade II (moderately differentiated)	4.53 (2.23–10.88)	<0.001	3.77 (1.79–9.25)	0.001
Grade III (poorly differentiated)	9.36 (4.63–22.38)	<0.001	6.84 (3.27–16.75)	<0.001
Grade IV (undifferentiated)	7.21 (2.67–20.93)	<0.001	5.07 (1.75–15.63)	0.003
**Primary site**				
Cervical esophagus	Reference		Reference	
Thoracic esophagus	0.85 (0.49–1.52)	0.572	0.79 (0.43–1.48)	0.446
Abdominal esophagus	1.35 (0.83–2.32)	0.245	1.55 (0.86–2.86)	0.153
Overlapping lesion of esophagus	2.02 (0.95–4.30)	0.068	1.34 (0.58–3.11)	0.494
**Histology**				
Adenocarcinoma	Reference		Reference	
Squamous cell carcinoma	0.89 (0.70–1.13)	0.359	0.99 (0.69–1.41)	0.946
Others	1.42 (0.79–2.48)	0.225	1.20 (0.61–2.29)	0.588
**Tumor size**				
0–21 mm	Reference		Reference	
22–47 mm	3.17 (2.22–4.62)	<0.001	3.34 (2.30–4.94)	<0.001
>48 mm	8.27 (5.89–11.86)	<0.001	8.56 (5.98–12.51)	<0.001

**Figure 9:**
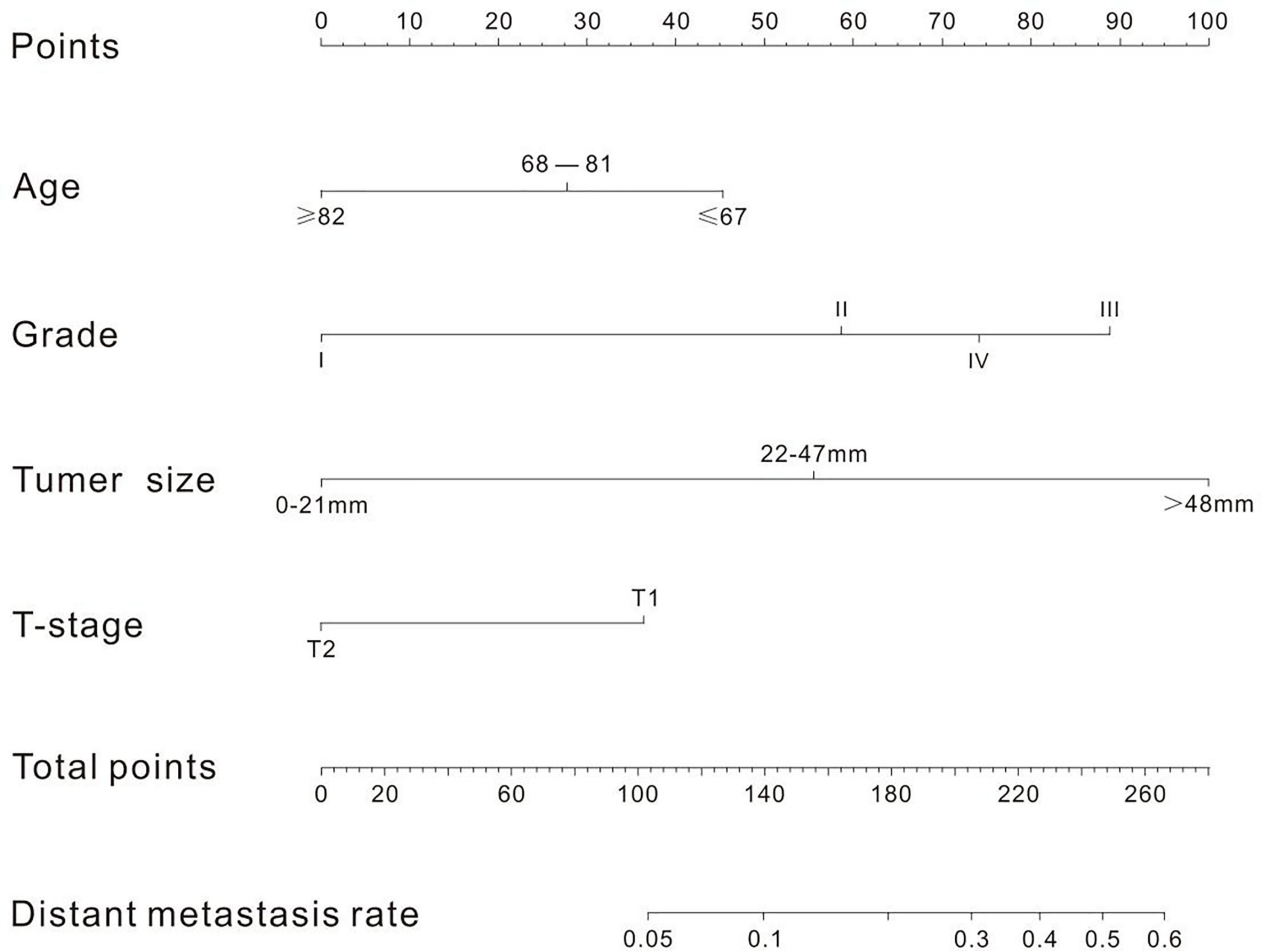
There are four factors in the nomogram. After taking values for these four factors (the "point" scale above), the total score is calculated, and the corresponding DM rate (the "total point" scale below) is obtained according to the total score.

**Figure 10:**
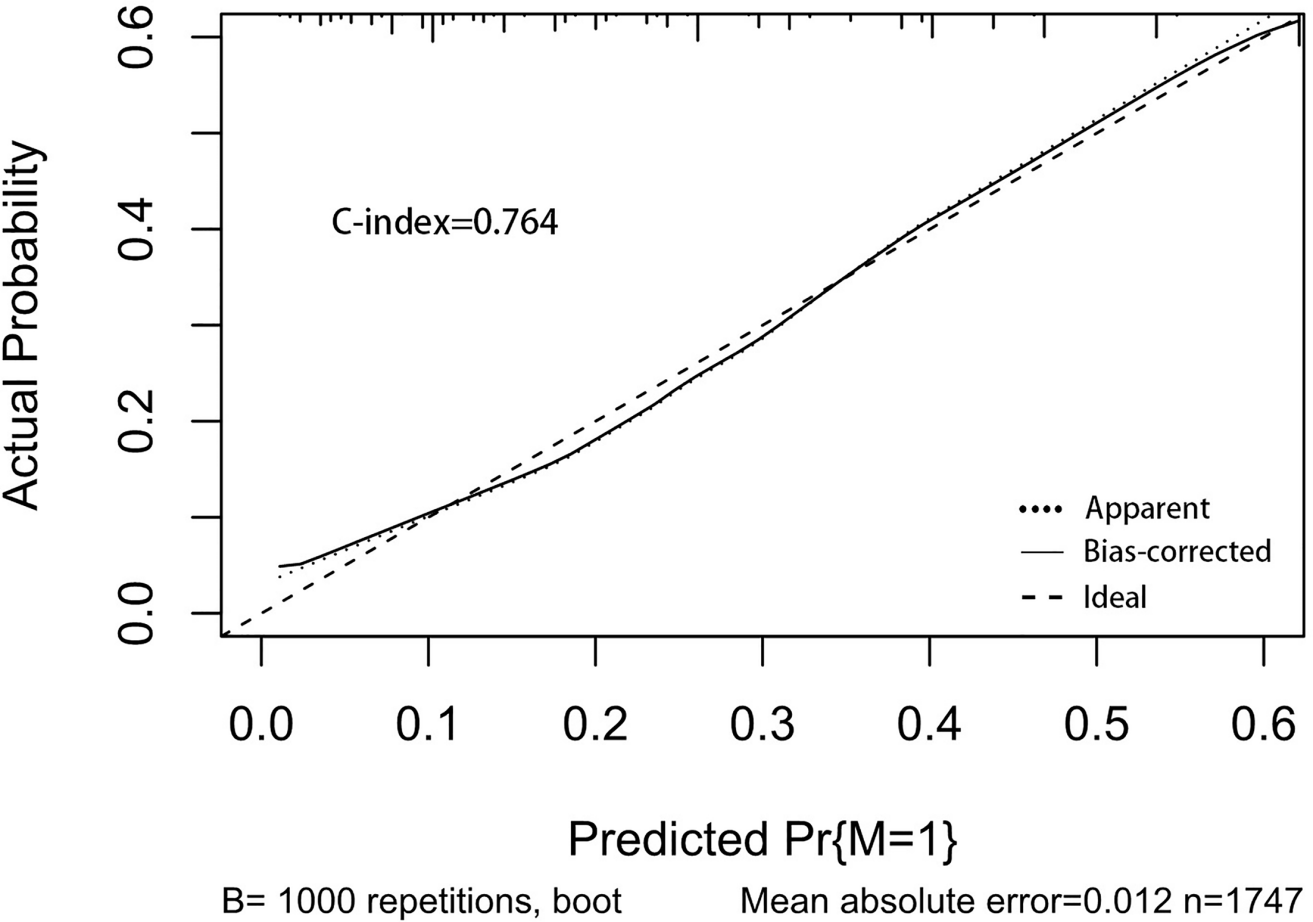
The calibration curve used to predict DM, with C-index at 0.764. The diagonal line indicates a coincidence between the actual and predicted DM probabilities, indicating that the probability predicted by the nomogram is very consistent with the actual observed values. The solid line is close to the diagonal line.

**Figure 11:**
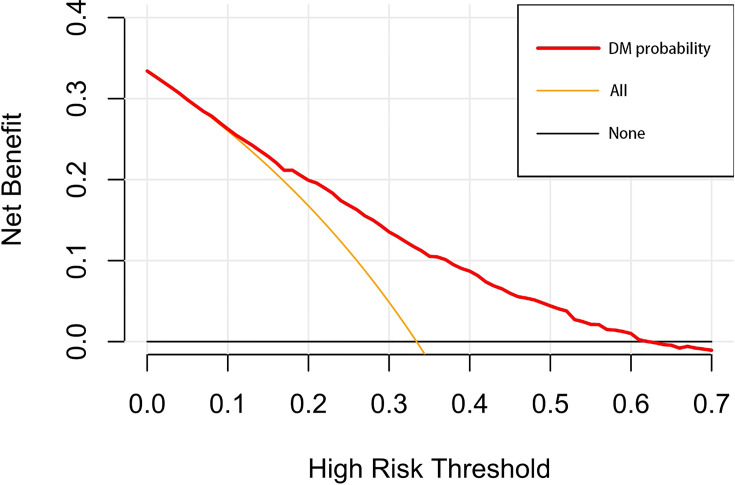
The decision curve draws a nomogram of the predicted DM. The x-axis represents the threshold probability and the y-axis represents the net benefit. The horizontal black line indicates that no patient has an extreme condition of DM, and the yellow line indicates that all patients have another extreme condition of DM.

**Figure 12:**
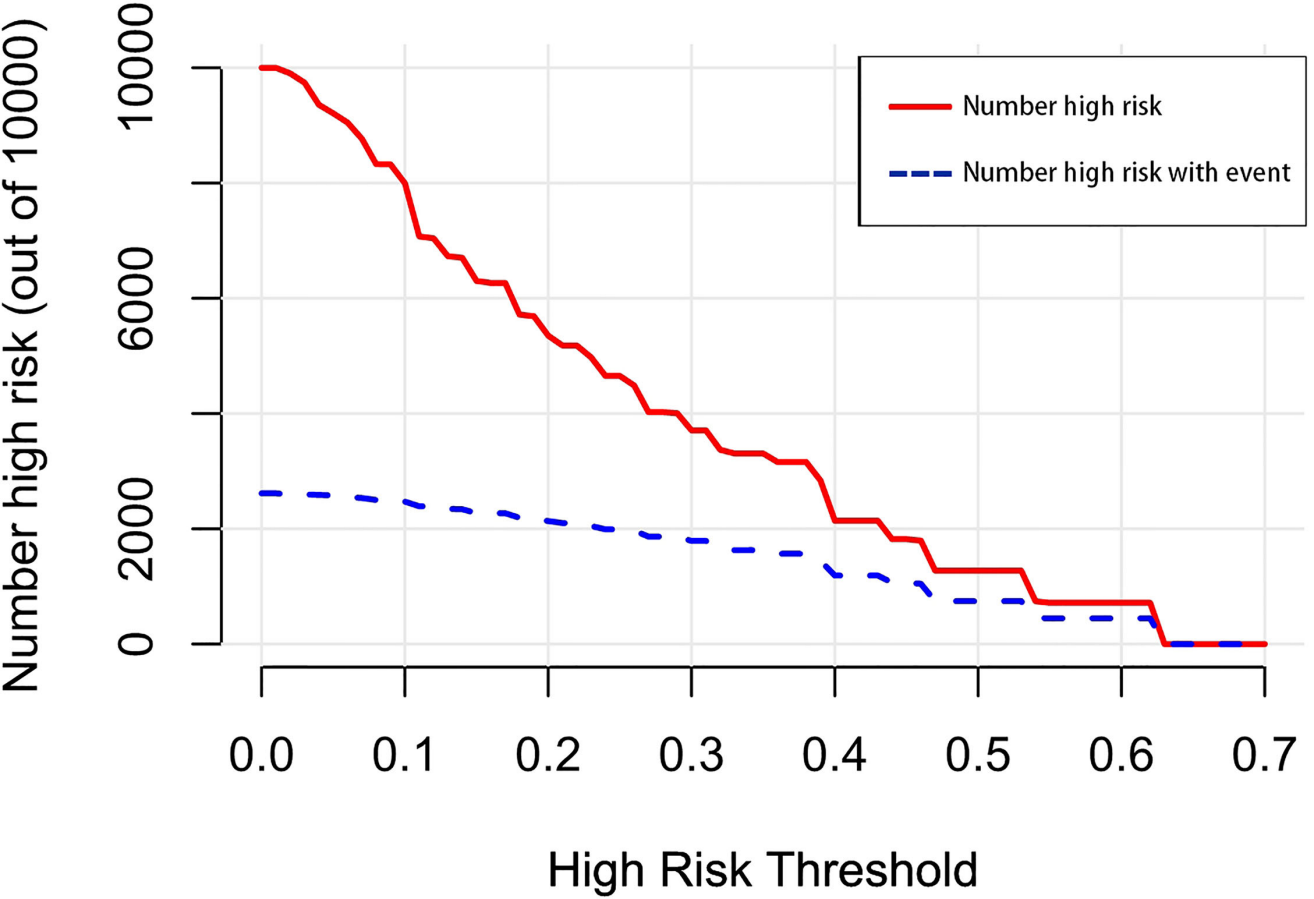
The number of high-risk patients and the number of high-risk patients with events are drawn with red solid lines and blue dotted lines to represent different threshold probabilities, respectively.

A nomogram was established to visually display the risk factors of DM (**Figure 9**). In addition, the exact scores of each factor in the nomogram are as shown in **Table 5**. From the perspective of score weight, tumor size was the most significant factor for influencing DM of T1–2 ESCA patients, followed by tumor grade, age, and T-stage. The calibration curve revealed that the nomogram had a strong resolution with the C-index of 0.764 (**Figure 10**). In addition, we conducted DCA and CIC on the DM nomogram (**Figures 11**, **12**), and the results showed that the DM nomogram was effective to predict DM in T1–2 ESCA patients in the threshold range of 0–0.27.

**Table 5:** Score of risk factors for nomograms.

Risk factors	Nomogram score		
	N	M	OS
**Age**			
≤67	58	45	0
68–81	35	28	23
≥82	0	0	47
**T-stage**			
T1	0	36	/
T2	58	0	/
**Sex**			
Male	/	/	14
Female	/	/	0
**Race**			
White	63	/	14
Black	61	/	40
Asian or Pacific Islander	100	/	0
American Indian/Alaska Native	0	/	63
**Grade**			
Grade I	0	0	/
Grade II	58	59	/
Grade III	78	89	/
Grade IV	66	74	/
**Primary site**			
Cervical esophagus	/	/	0
Thoracic esophagus	/	/	24
Abdominal esophagus	/	/	19
Overlapping lesion of esophagus	/	/	61
**Histology**			
Adenocarcinoma	/	/	0
Squamous cell carcinoma	/	/	7
Others	/	/	34
**Tumor size**			
0–21 mm	0	0	0
22–47 mm	33	55	27
>48 mm	67	100	40
**Surgery (primary site)**			
None	/	/	100
Yes	/	/	0
**N-stage**			
NO	/	/	0
N1	/	/	18
**M-stage**			
M0	/	/	0
M1	/	/	49

### Survival Analysis of ESCA Patients With LNM and DM

The effects of LNM and DM on survival were calculated by Kaplan–Meier and gray methods. OS was associated with LNM (SHR = 1.511, 95% CI = 0.662–1.731, *p* < 0.0001) and DM (SHR = 3.214, 95% CI = 2.852–3.622, *p* < 0.0001) (**Supplementary Figures 1**, **2**). LNM (HR = 2.127, 95% CI = 1.613–2.805, *p* < 0.001) and DM (HR = 11.667, 95% CI = 7.326–18.580, *p* < 0.001) were also associated with CSS as revealed by the Gray method (**Supplementary Figures 3**, **4**).

### Risk Factors and Nomogram of OS

Based on the multivariate Cox proportional hazards regression model, prognostic factors for OS of T1–2 ESCA patients were identified. To more intuitively display the results of the multivariable Cox proportional risk model, forest plots were depicted in **Supplementary Figure 5**. The results showed that there were 9 prognostic factors, including age, race, sex, histology, primary site, tumor size, N-stage, M-stage, and surgery, while tumor grade and T-stage were not correlated with OS. The prognosis of patients aged 68–81 years (HR = 1.29, 95% CI = 1.15–1.46, *p* < 0.001) or ≥82 years (HR = 1.72, 95% CI = 1.44–2.05, *p* < 0.001) was worse than those aged younger than 67 years. Concerning race, black patients suffered a worse prognosis than did white patients (HR = 1.38, 95% CI = 1.11–1.68, *p* = 0.003). Female patients had a better prognosis than males (HR = 0.87, 95% CI = 0.76–1.01, *p* = 0.04). Compared with T1–2 ESCA patients with the origin of the cervical esophagus, the prognosis of those with the origin of the thoracic esophagus (HR = 1.32, 95% CI = 1.02–1.70, *p* = 0.03) and overlapping lesion of esophagus (HR = 2.12, 95% CI = 1.45–3.10, *p* < 0.001) was significantly worse. The larger the primary tumor size, the worse the prognosis. Compared with patients with a tumor size of 0–21 mm, T1–2 ESCA patients with a tumor size of 22–47 mm (OR = 1.35, 95% CI = 1.16–1.58, *p* < 0.001) and >48 mm (OR = 1.54, 95% CI = 1.32–1.80, *p* < 0.001) was worse. Undoubtedly, the prognosis of patients with LNM (OR = 1.22, 95% CI = 1.09–1.38, *p* < 0.001) and DM (OR = 1.71, 95% CI = 1.49–1.97, *p* < 0.001) was worse than of those without metastases. Based on the Cox regression model, the 3-, 5-, and 10-year OS prognostic nomograms are as shown in **Supplementary Figure 6**. By adding up the scores of each factor, the probability of 3-, 5-, and 10-year OS in T1–2 ESCA patients could be calculated. The C-index was 0.740, and the correction curve showed that the predicted results were consistent with the actual situation (**Supplementary Figures 7, 8, 9**).

## Discussion

T1-2 ESCA is characterized as the invasion of the lamina propria, muscularis mucosa, submucosa, or muscularis propria, rather than the esophageal fibrous membrane (2). In the present study, about 49% of newly diagnosed T1–2 ESCA patients did not have LNM and DM, and about 33% of them had LNM, but no DM. Moreover, about 26% of T1–2 ESCA patients had DM. Due to the different statues of LNM and DM, the therapeutic strategies and corresponding prognoses of T1–2 ESCA patients were individualized. At present, pathological biopsy is still the gold standard for the diagnosis of LNM and DM in ESCA patients. Although simple examinations like PET-CT can be used to assess LNM and DM in ESCA patients, its application is limited due to high cost, false-negative rate, and false-positive rate (18). Therefore, a non-invasive and effective method to evaluate the presence of LNM and DM in ESCA patients is urgently needed. According to the prediction results of the model, further examination and therapeutic strategies can be selected more reasonably.

In recent years, a growing number of studies have focused on the prediction models of human diseases, although deficiencies and limitations exist. Previous studies established Cox regression models based on logistic regression analysis, but these models have low prediction ability and cannot be used in clinical practice (19, 20). As a new form of prediction models, a nomogram can directly visualize the predicted LNM and DM, which provides a reference for further examinations and clinical decision-making. At present, many nomograms can be used to predict the diagnosis and prognosis of cancers, but there are many problems like the sample size (21), low C-index and the prediction accuracy of the model (22), insufficient inclusion and exclusion criteria (23), lack of cutoff values (24–26), and latest evidence (27). To our knowledge, this is the only published study to establish a nomogram to predict the incidence and survival rate of LNM and DM in T1–2 ESCA patients by analyzing latest cancer data from 1975 to 2018 in the SEER database. The included subjects were divided into group N (T1-2N0-3M0 ESCA patients for predicting LNM) and group M (T1-2N0-3M0-1 ESCA patients for predicting DM). Three nomograms were established and validated to predict LNM, DM, and OS in T1–2 ESCA patients. The LNM nomogram included five factors, namely, age, race, grade, tumor size, and T-stage. The DM nomogram included four factors age, T-stage, grade, and tumor size. The nomogram of survival rate included 9 factors age, race, sex, histology, primary site, tumor size, N-stage, M-stage, and surgery. The C-indexes of LNM nomogram, DM nomogram, and prognostic nomogram were 0.737, 0.764, and 0.740, respectively, indicating their good clinical value.

Previous studies have shown that age, depth of tumor invasion, tumor size, and grade are related to the risk of LNM in ESCA patients (4). Our findings also revealed that T1–2 ESCA patients with an old age had a lower risk of LNM, which may be attributed to low tumor differentiation in young cancer patients prone to escape immune surveillance. This speculation lacks conclusive data and needs further exploration. T2 ESCA patients had a higher risk of LNM than those with T1. In addition, T1–2 ESCA patients with a larger tumor size had a higher risk of LNM than those with a smaller cancer lesion. In the relationship between grade and LNM, the LNM risks of moderately differentiated cancer, poorly differentiated cancer, and undifferentiated cancer were 2.79, 4.06, and 3.25, respectively. The overall results were also consistent with our conventional cognition. A higher degree of differentiation indicated lower malignant level and possibility to metastasize. However, the proportion of undifferentiated LNM was lower than that in poorly differentiated patients. We considered that a small sample size (41 cases) and early-stage ESCA (T1–2) may cause inconsistent findings. Similar results were obtained showing that T1–2 ESCA patients with an old age had a lower risk of DM than did young patients. A previous study has shown that age is an independent predictor of metastatic organs in cancer patients, and young patients are more prone to have a metastasis (28). Advanced T-stage and large tumor size were both risk factors of DM in T1–2 ESCA patients. In the relationship between grade and DM, the DM risk of moderately differentiated cancer, poorly differentiated cancer, and undifferentiated cancer was 3.77, 6.84, and 5.07, respectively, which was similar to that in the LNM nomogram. Surprisingly, LNM and DM were not correlated with primary site, histology, and sex, which were inconsistent with previous findings (29, 30). In the established OS nomogram, there were 9 factors, including age, race, sex, histology, primary site, tumor size, N-stage, M-stage, and surgery, while it was not related with T1/T2 and grade.

In addition, we found that LNM and DM of T1–2 ESCA were associated with tumor-specific and non-tumor-specific death. Since all clinical data were screened out from 1,747 eligible patients with the mean follow-up for 70 months recorded in the public database, the data and statistical results were convincing.

This study had some limitations. First of all, it was a population-based retrospective analysis lacking prospective data for verification. Secondly, the database had insufficient information about high-risk lifestyle factors (e.g., large consumption of alcohol, eat high-temperature food or pickled food), tumor markers, imaging examination, important molecular factors (PD-1/PD-L1 gene status), metastasis sites, etc. They are believed as important factors for predicting LNM, DM, and prognosis of T1-2 ESCA which should be further explored. Thirdly, sarcoma and GIST are also malignant tumors with ICDO/3. However, there are other malignant epithelial tumors, so the prediction model established in this paper is not applicable to “Sarcoma and GIST.” Finally, our data were only from the United States population and the sample size was relatively small. In the future, multicenter data with a large sample size and population in different races should be analyzed to validate our conclusions.

Collectively, three nomograms were established based on analysis of independent risk factors for T1–2 ESCA patients from downloaded data in the online database for predicting LNM, DM, and OS. Involved factors in nomograms can be easily obtained from clinical records, suggesting the convenience of applying established nomograms in clinical practice. Combined with other clinical data, the established nomograms are expected to assist physicians to make better diagnosis, individualized treatment, and follow-up management for T1–2 ESCA patients.

## Data Availability Statement

The original contributions presented in the study are included in the article/**Supplementary Material**. Further inquiries can be directed to the corresponding author.

## Author Contributions

YQ, SW, JL, and ZF designed the study. LT, GX, YW, and JC extracted and analyzed the data. YQ and CL wrote and edited the manuscript. The authors were ranked according to their contributions. YQ and SW contributed equally to this work and should be considered as co-first authors.

## Funding

This study was funded by the Key Research Department Project of Oncology Department of Tongde Hospital of Zhejiang Province; construction project of inheritance studio for famous and old traditional Chinese medicine experts chaikequn in Zhejiang Province (GZS2017001); Zhejiang Provincial Medical and Health Science and Technology Project (2021417777); Zhejiang Provincial Chinese Medicine Science and Technology Project (2021ZB059); and Zhejiang Provincial Natural Science Foundation (LQ19H030004).

## Conflict of Interest

The authors declare that the research was conducted in the absence of any commercial or financial relationships that could be construed as a potential conflict of interest.

## Publisher’s Note

All claims expressed in this article are solely those of the authors and do not necessarily represent those of their affiliated organizations, or those of the publisher, the editors and the reviewers. Any product that may be evaluated in this article, or claim that may be made by its manufacturer, is not guaranteed or endorsed by the publisher.

## Supplementary Material

Supplemenatary Figure 1 The effect of LNM on OS. There is a significant difference in survival time between the two groups. The data below indicate the number of patients who survived after each time period with or without LNM.

Supplementary Figure 2 The effect of DM on OS. There is a significant difference in survival time between the two groups. The data below indicate the number of patients who survived after each time period with or without DM.

Supplementary Figure 3 There is a significant difference between CSD with LNM and without LNM. There is no significant difference between NCSD with LNM and without LNM.

Supplementary Figure 4 There is a significant difference between CSD with DM and without DM. There is no significant difference between NCSD with DM and without DM.

Supplementary Figure 5 Forest plot depicting the significance of multivariate Cox proportional hazard regression prognostic factors of OS in T1-2 ESCA patients. Among them, the gray shading group is the control group.

Supplementary Figure 6-9 Nomogram (6) and calibration curve (7, 8, 9). There are 9 factors in **Supplementary Figure 6**. After taking values for these 9 factors (the “point” scale above), the total score is calculated, and the corresponding survival prediction is obtained according to the total score (the “total point” scale below). 7, 8, and 9 The calibration curves for predicting 1-, 3-, and 5-year OS, respectively, and the C-index is 0.740. The diagonal indicates a coincidence between the actual and predicted OS probabilities. When the solid line is close to the diagonal line, it shows that the probability predicted by the nomogram is very consistent with the actual observed value.
