# Supplementary figures and images for: A Population-Based Study: How to Identify High-Risk T1–2 Esophageal Cancer Patients?
Source: Front Oncol. 2021 Dec 13;11:766181. doi: 10.3389/fonc.2021.766181 (PMC8710781; doi:10.3389/fonc.2021.766181)

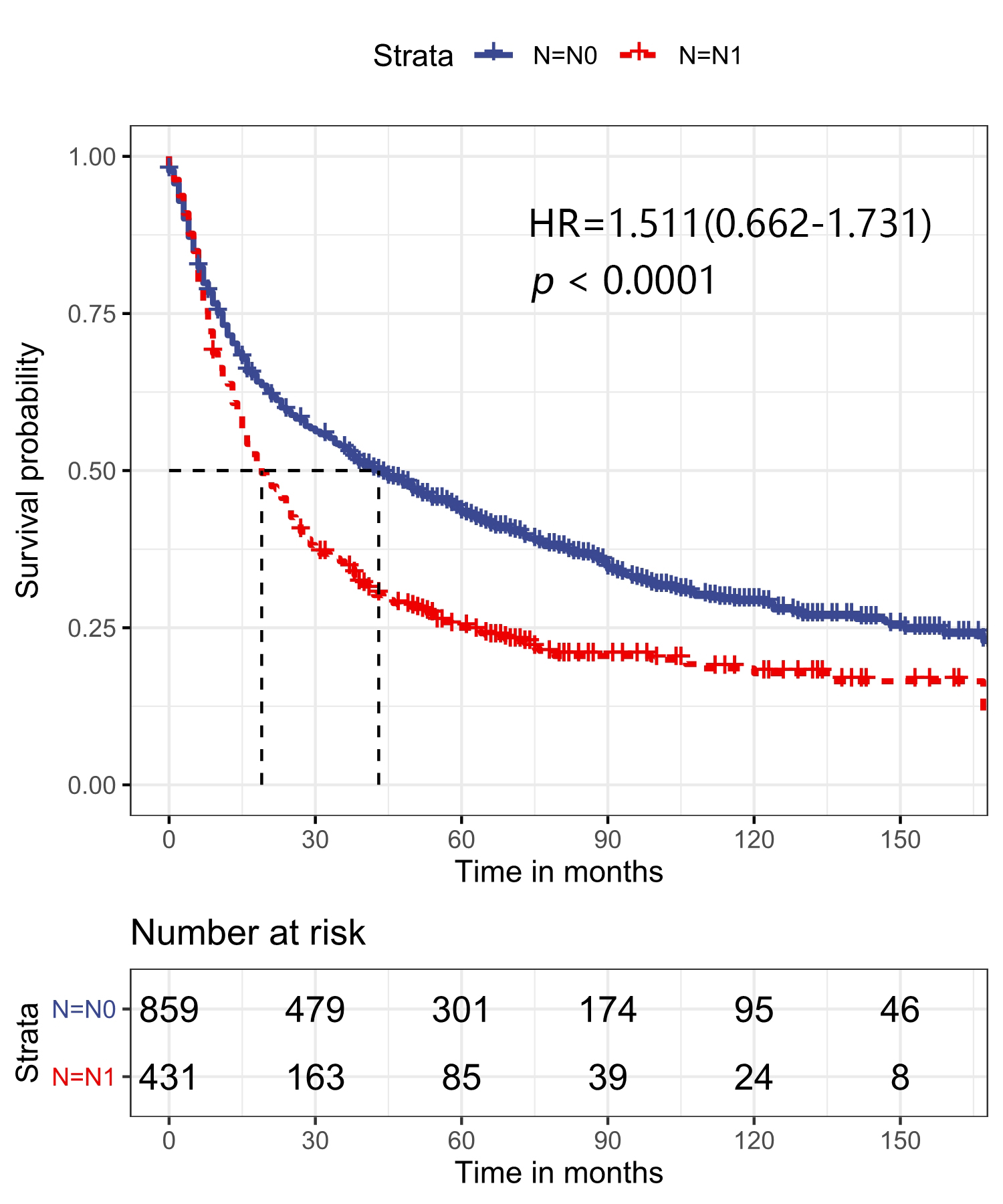

Supplement: Supplemenatary Figure 1 — The effect of LNM on OS. There is a significant difference in survival time between the two groups. The data below indicate the number of patients who survived after each time period with or without LNM. [file Image_1.jpeg]

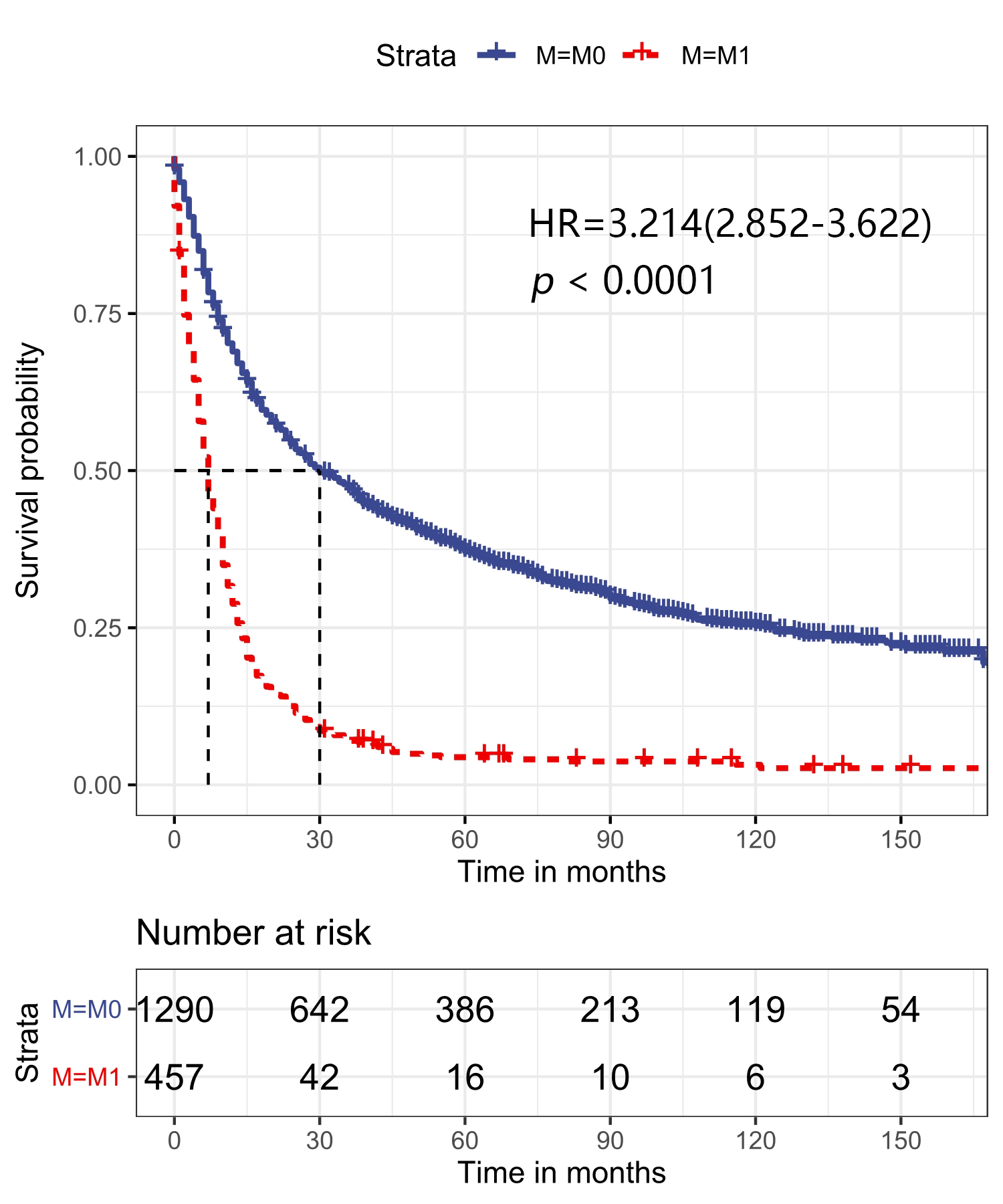

Supplement: Supplementary Figure 2 — The effect of DM on OS. There is a significant difference in survival time between the two groups. The data below indicate the number of patients who survived after each time period with or without DM. [file Image_2.jpeg]

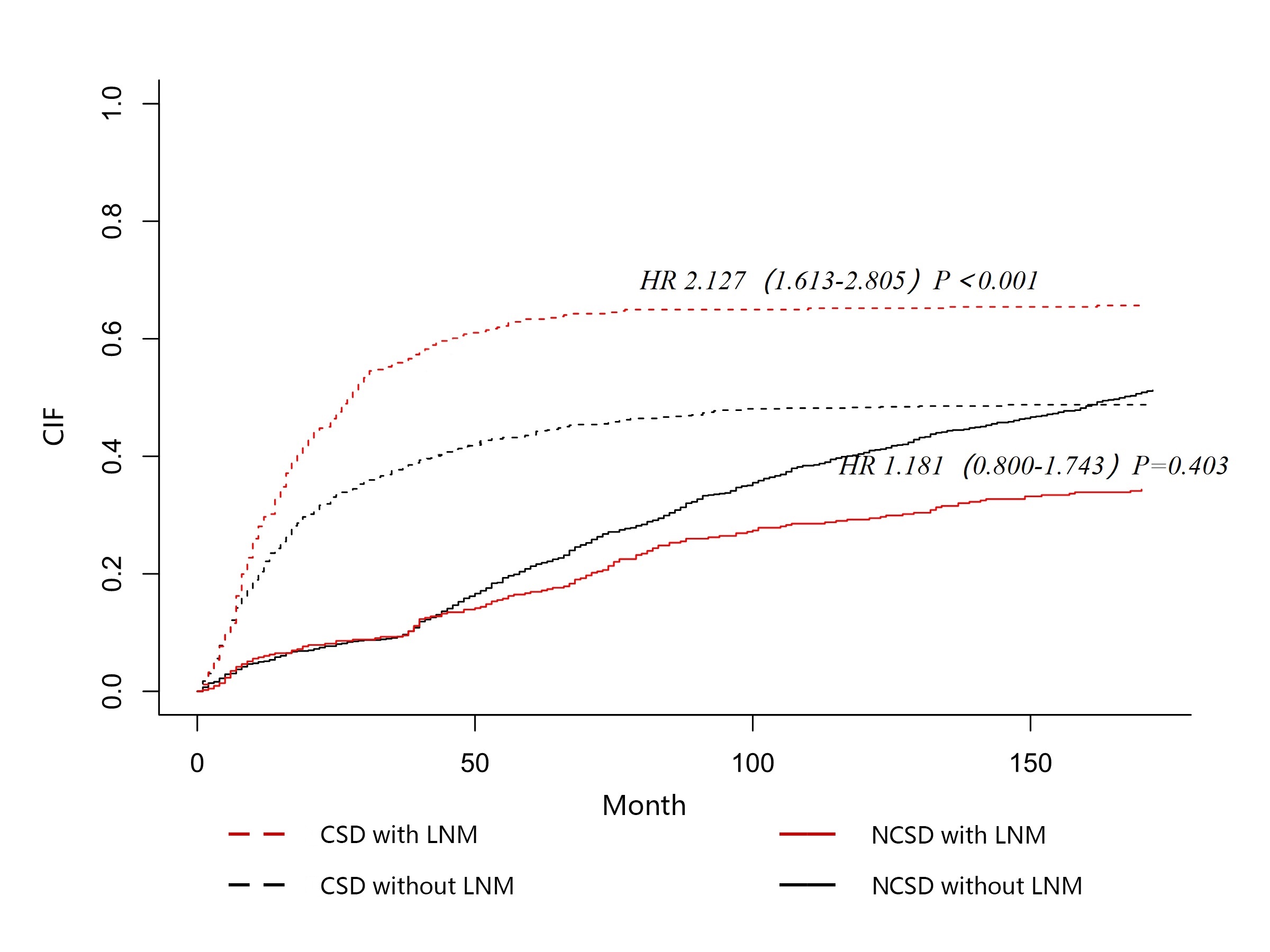

Supplement: Supplementary Figure 3 — There is a significant difference between CSD with LNM and without LNM. There is no significant difference between NCSD with LNM and without LNM. [file Image_3.jpeg]

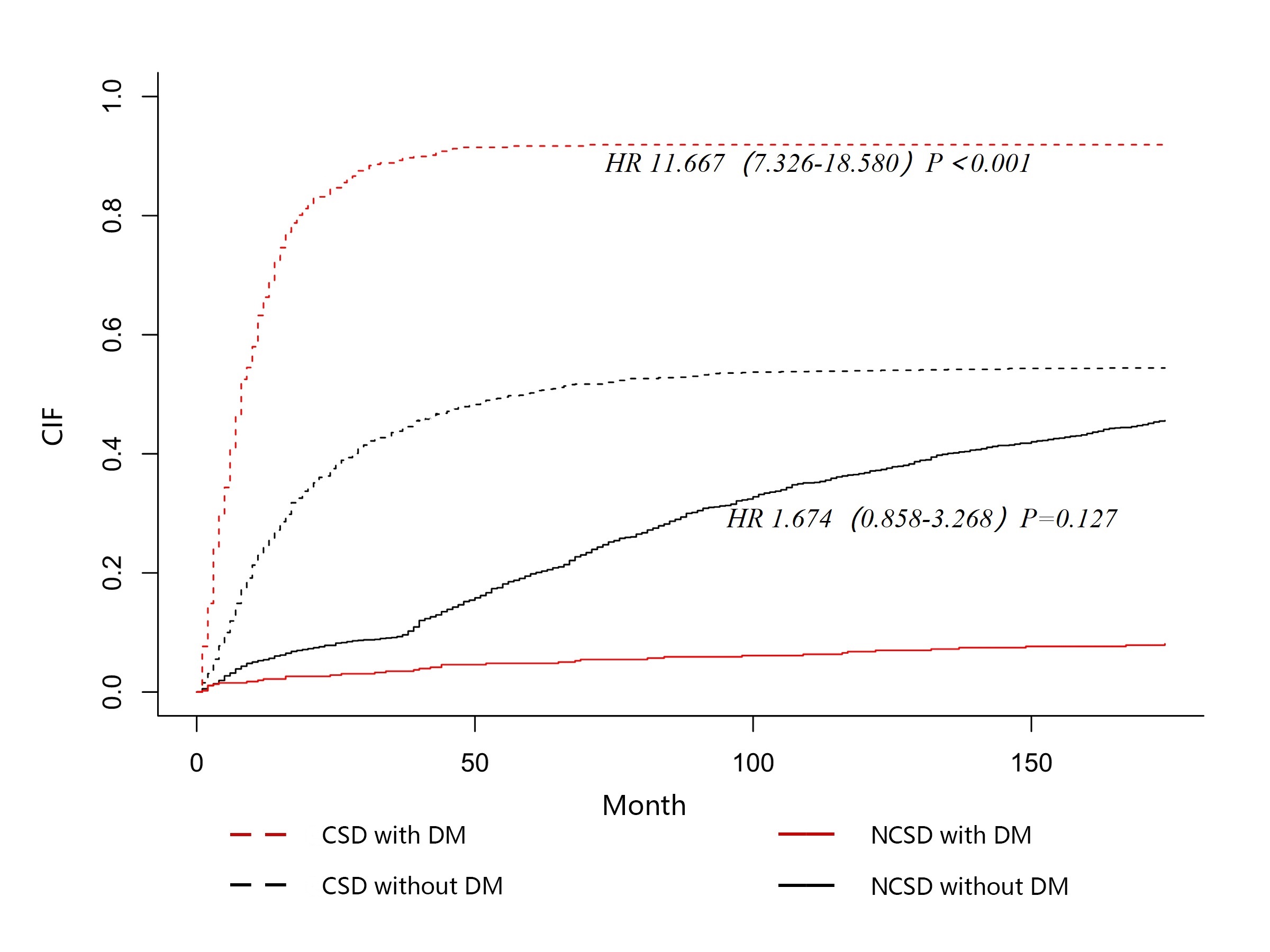

Supplement: Supplementary Figure 4 — There is a significant difference between CSD with DM and without DM. There is no significant difference between NCSD with DM and without DM. [file Image_4.jpeg]

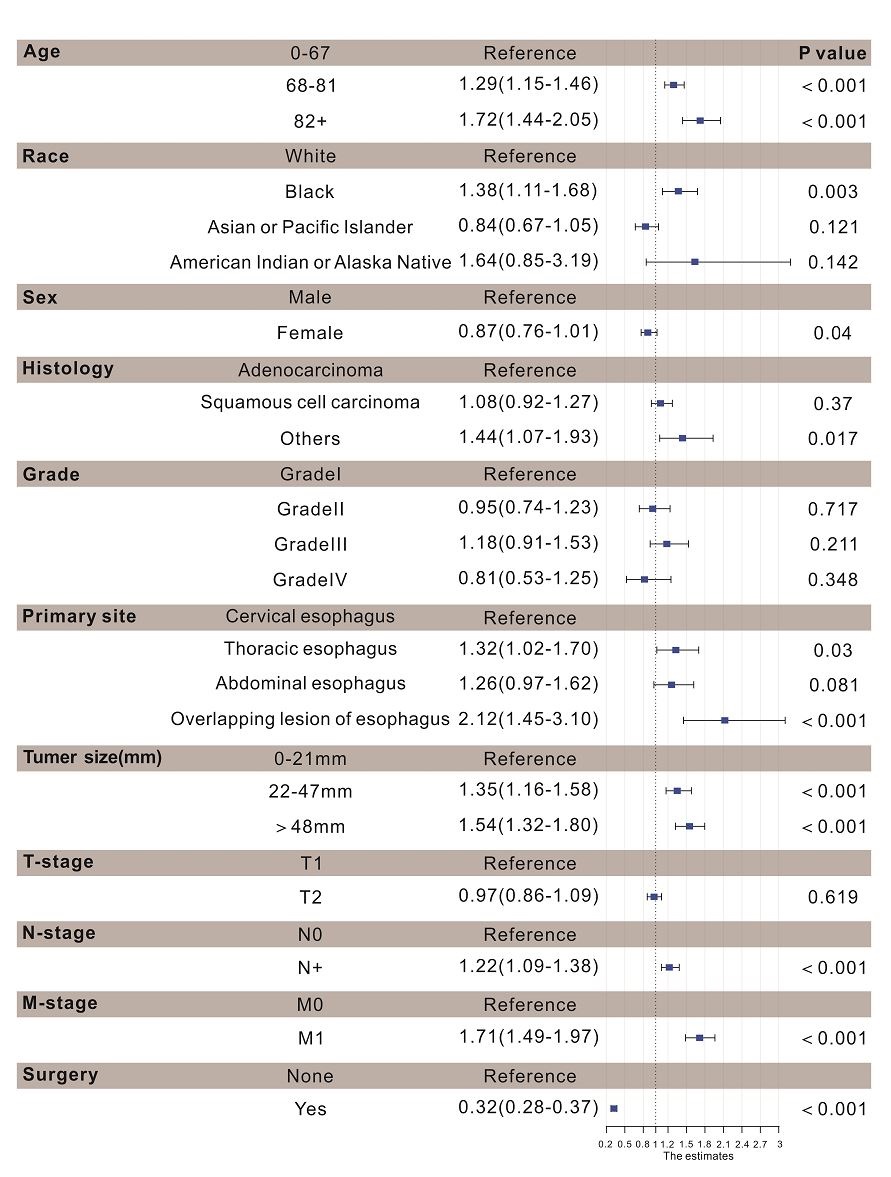

Supplement: Supplementary Figure 5 — Forest plot depicting the significance of multivariate Cox proportional hazard regression prognostic factors of OS in T1-2 ESCA patients. Among them, the gray shading group is the control group. [file Image_5.jpeg]

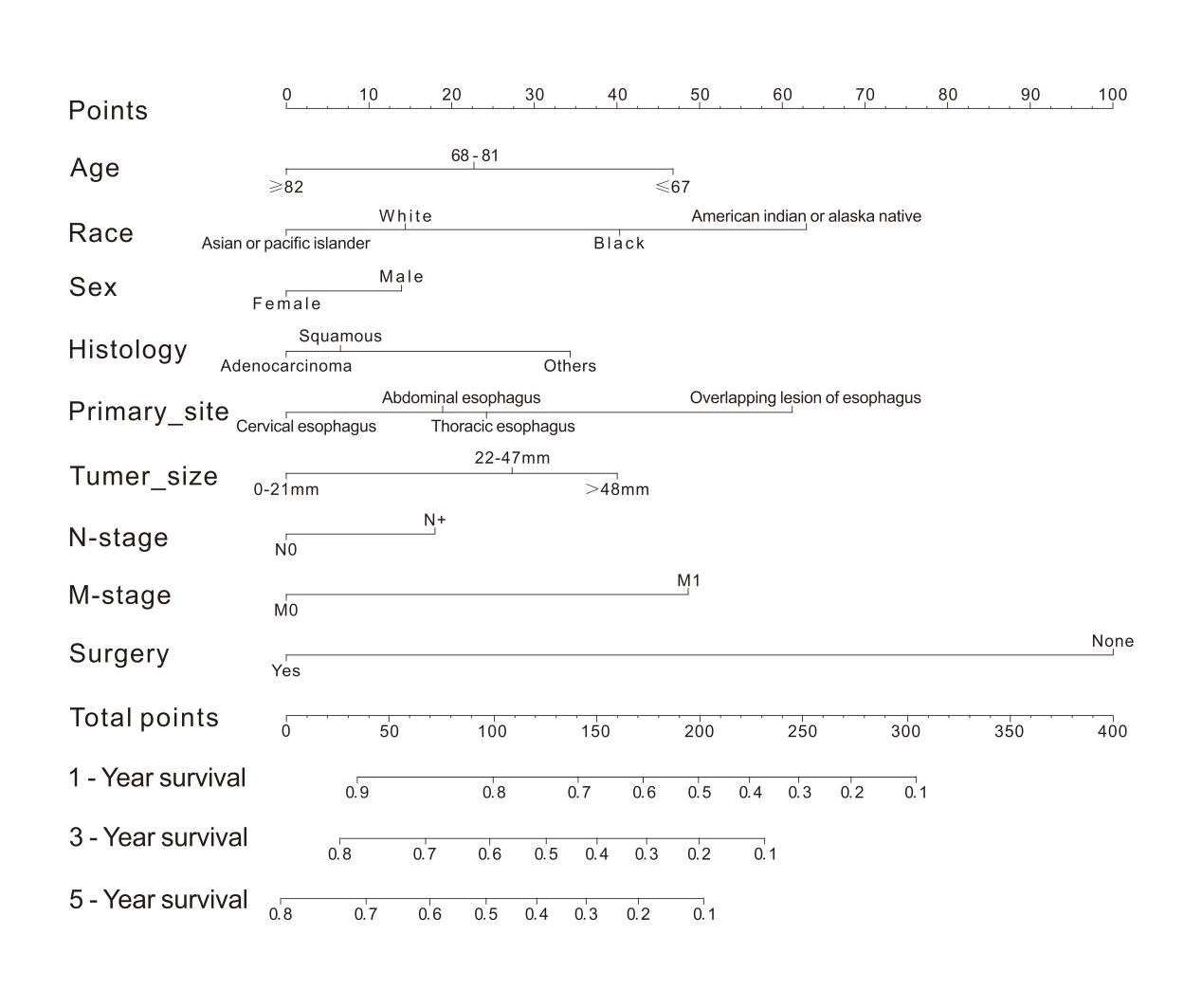

Supplement: Supplementary Figure 6-9 — Nomogram (6) and calibration curve (7, 8, 9). There are 9 factors in Supplementary Figure 6 . After taking values for these 9 factors (the “point” scale above), the total score is calculated, and the corresponding survival prediction is obtained according to the total score (the “total point” scale below). 7, 8, and 9 The calibration curves for predicting 1-, 3-, and 5-year OS, respectively, and the C-index is 0.740. The diagonal indicates a coincidence between the actual and predicted OS probabilities. When the solid line is close to the diagonal line, it shows that the probability predicted by the nomogram is very consistent with the actual observed value. [file Image_6.jpeg]

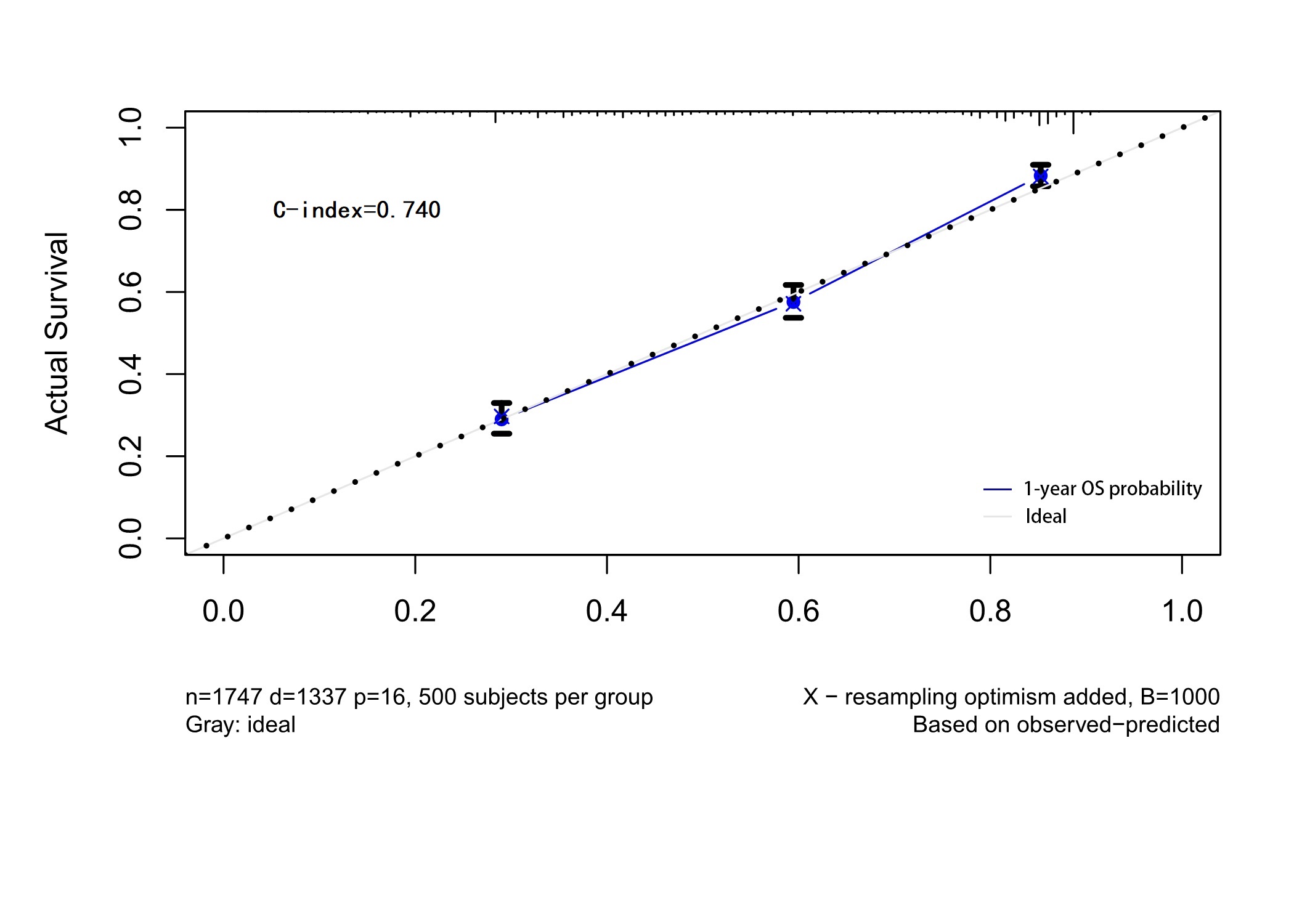

Supplement: Supplementary file 7 [file Image_7.jpeg]

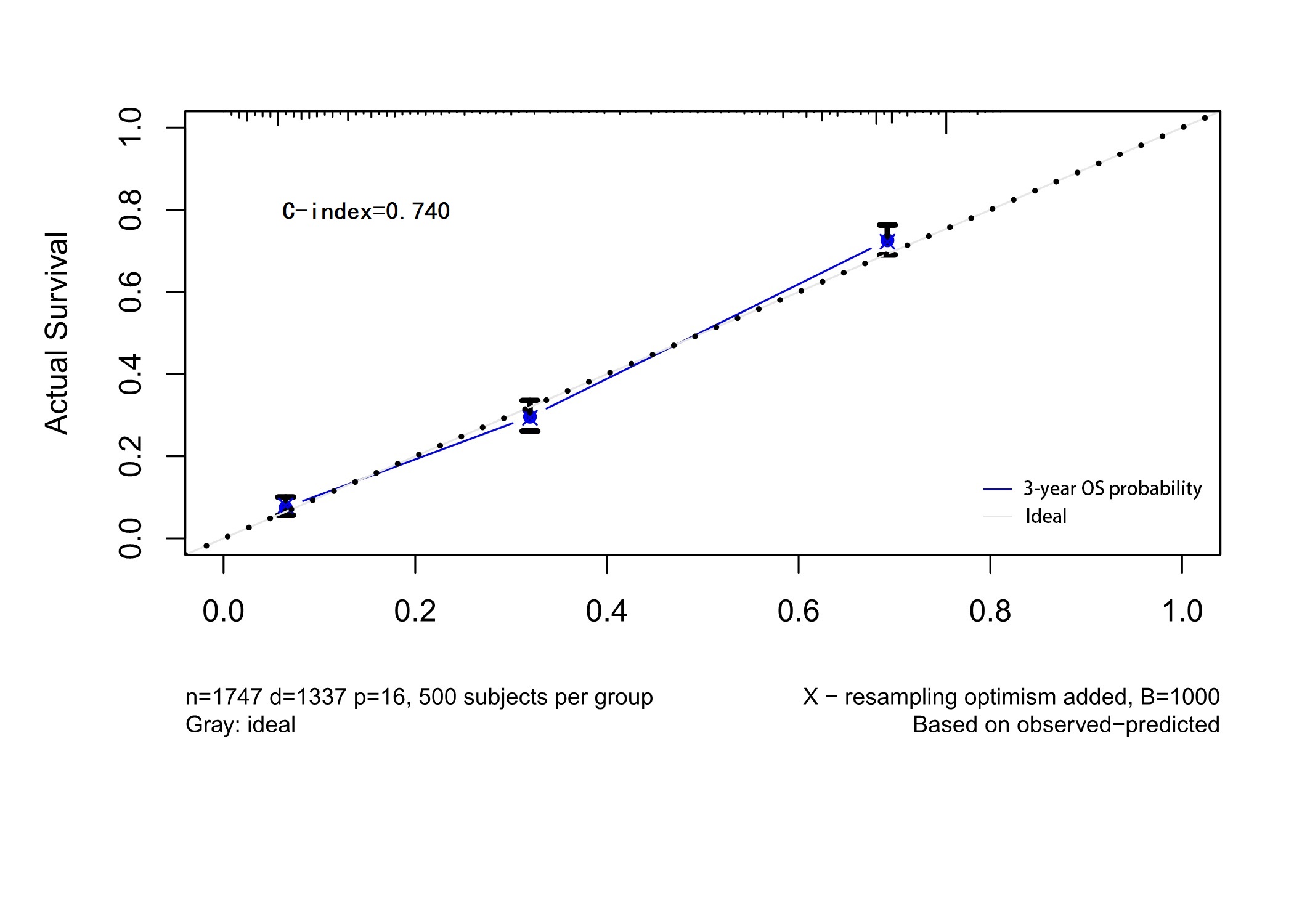

Supplement: Supplementary file 8 [file Image_8.jpeg]

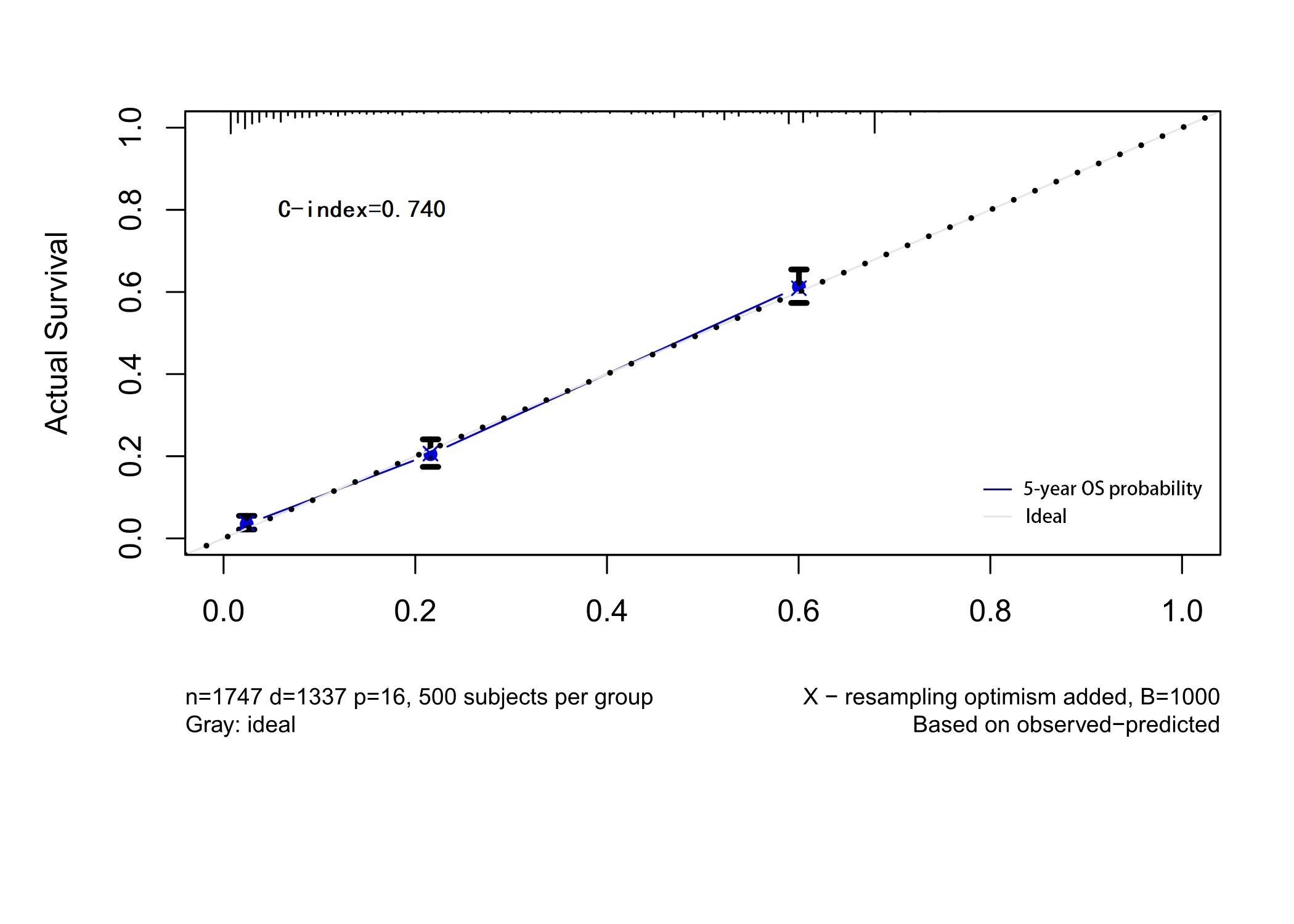

Supplement: Supplementary file 9 [file Image_9.jpeg]
